# Patient reported symptoms associated with quality of life during chemo‐ or immunotherapy for bladder cancer patients with advanced disease

**DOI:** 10.1002/cam4.2958

**Published:** 2020-03-10

**Authors:** Gry A. Taarnhøj, Christoffer Johansen, Henriette Lindberg, Ethan Basch, Amylou Dueck, Helle Pappot

**Affiliations:** ^1^ Department of Oncology University Hospital of Copenhagen Rigshospitalet Copenhagen Denmark; ^2^ Department of Oncology University Hospital of Copenhagen Herlev Hospital Herlev Denmark; ^3^ Lineberger Comprehensive Cancer Center University of North Carolina Chapel Hill NC USA; ^4^ Section of Biostatistics Mayo Clinic Scottsdale AZ USA

**Keywords:** chemotherapy, immunotherapy, Patient‐reported outcomes, quality of life, urinary bladder neoplasms

## Abstract

**Background:**

Bladder cancer (BC) patients with advanced disease have poor outcomes. The use of patient‐reported outcomes (PROs) could lead to improvements in symptom management and hence quality of life (QoL). The aim of this study is to report correlations between selected PROs and QoL and thus to present symptoms that influence QoL. Identification of these symptoms during treatment can lead to earlier symptom management and thus secure improvements in QoL.

**Methods:**

BC patients in chemo‐ or immunotherapy for locally advanced or metastatic disease reported weekly PROs for the duration of their treatment. The PROs included EORTC QLQ‐C30 and QLQ‐BLM30 and 45 selected PRO‐CTCAE items. Spearman's correlation analysis was performed for all PRO‐CTCAE items and QLQ‐C30 global QoL and subdomains.

**Results:**

In this study, 78 BC patients reported 724 questionnaires. Spearman's analysis showed significant correlations between almost all PRO‐CTCAE items and the expected domain of QoL. The PRO‐CTCAE items with the strongest correlations with QoL were anxiety (F, frequency item) and emotional function (*r*_s_ = −0.603, *P* < .0001), concentration (S, severity item) and cognitive function (*r*_s_ = −0.704, *P* < .0001), discouraged (F) and emotional function (*r*_s_ = −0.659, *P* < .0001), fatigue (S) and role function (*r*_s_ = −0.659, *P* < .0001) and sad (F) and emotional function (*r*_s_ = −0.711, *P* < .0001). The weakest correlations were found for the PRO‐CTCAE items urinary frequency, incontinence and urge, all with variations in the direction and significance of the correlations.

**Conclusions:**

This study delivers information on which PROs may influence QoL for patients in clinical trials or daily clinic. Psychological issues have a strong impact on QoL and should be dealt with during treatment to secure the best possible QoL for BC patients.

## Graphical abstract

For bladder cancer patients, psychological items have a strong impact on the quality of life while urinary items have a weak impact on the quality of life. Toxicity reporting from clinical trials and supportive care in daily practice may be incomplete and not reflective of the full picture of symptomatology for bladder cancer patients.

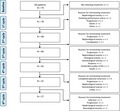

## 1 INTRODUCTION

Bladder cancer patients with muscle‐invasive or metastatic disease adhere poorly to treatment, have a feeble prognosis and are thus in urgent need of better treatment options.^1^, ^2^, ^3^, ^4^ One may hypothesize that better supportive care and symptom monitoring during treatment would increase adherence to treatment thereby improve clinical outcomes. Recently published studies have shown that the use of patient‐reported outcomes (PROs) actively during treatment represents a way of achieving such outcomes.^5^, ^6^, ^7^, ^8^, ^9^ The Patient‐Reported Outcomes version of the Common Terminology Criteria for Adverse Events (PRO‐CTCAE) has been proposed for use in clinical trials as a supplement to the widely used CTCAE but is, however, still not part of standard reporting in clinical trials or daily clinic.^10^, ^11^ Instead, measurement of quality of life (QoL) is widely used in clinical trials but this concept is challenged when distinguishing between two treatment arms in which the toxicity is expected to be different but is reported equal in terms of quality of life.^12^, ^13^ Traditionally, clinical trials in the bladder cancer population have reported substantial urinary, bowel, and sexual symptoms yet often no differences in global QoL are reported during follow‐up.^14^, ^15^, ^16^, ^17^ One may thus question whether the measures used are appropriate to show a difference in QoL for this specific population and whether the overall symptom burden correlates with QoL and its subdomains. Thereby questioning if the measures applied are reporting what actually matters to the patients.

During the validation of the PRO‐CTCAE, the European Organisation for Research and Treatment of Cancer (EORTC) general core questionnaire QLQ‐C30 was used as an anchor to test the correlations between the PRO‐CTCAE symptoms with the expected direction of QoL in the QLQ‐C30.^18^ The validation process confirmed the expected correlation between the two instruments and direction of QoL reflecting changes in symptom severity in the anticipated direction. Although extensive, this work did however not report specifically on bladder cancer patients and only few of these patients were included in the study. We may therefore not know which PRO‐CTCAE items have an impact on QoL specific for this patient group and hence where best to put efforts into supporting bladder cancer patients during treatment with the aim of improving QoL.

The aim of this study is therefore to report correlations between PRO‐CTCAE items, total PRO‐CTCAE symptom burden and QoL in the bladder cancer population during antineoplastic treatment and thus to describe which symptoms that are associated with QoL. In perspective, we are interested in directing the supportive care in the right direction for this patient group thereby securing focus on timely symptom management and the impact of such on QoL and clinical outcomes. At the same time, we hope that this information on pivotal symptomatic items for the bladder cancer population could be helpful when designing clinical trials regarding which items are relevant to this population to reflect the total toxicity burden during treatment and its effect on QoL.

## 2 METHODS

### 2.1 Participants

Data for this study were obtained from two prospective clinical studies (study 1 and 2) in patients with locally advanced or metastatic bladder cancer receiving chemo‐ or immunotherapy at two university hospitals (H1 and H2), Copenhagen, Denmark. The purposes of these two studies were to collect PROs longitudinally, report clinical outcomes (study 1&2) and evaluate electronic PRO completion (study 2), data on these issues will be reported elsewhere. Study 1 was conducted at H1 with both paper and electronic completion of questionnaires whereas study 2 was conducted at H1 and H2 with only electronic capture of PROs. Criteria for inclusion in both studies were as follows:

- Bladder cancer (stages T2‐)
- Initiating treatment with chemotherapy (combination cisplatin‐gemcitabine, carboplatin‐gemcitabine or single agent vinflunine) or immunotherapy (pembrolizumab) as the standard neoadjuvant or metastatic treatment.
- Able to read Danish.
- No serious cognitive impairment as evaluated by the treating team of physician and/or nurse.

### 2.2 Questionnaires

In both studies, the patients completed the same questionnaires weekly by paper or electronically through the Ambuflex software system in a web‐browser.^19^ The questionnaires included the following: the EORTC QLQ‐C30, EORTC QLQ‐BLM30, the Hospital Anxiety and Depression Scale (HADS), and 45 specifically chosen PRO‐CTCAE items for this population. The PRO‐CTCAE items were chosen through a mixed methods approach inspired by Nissen et al^20^ and described in detail in a separate paper.^21^ The total number of weekly questions amounted to 158. Completion of questionnaires commenced after written informed consent at day 1 of treatment (baseline). Every week, all of the above questionnaires were completed simultaneously throughout the course of treatment enabling PRO‐CTCAE items to be aligned with the response to the EORTC QLQ‐C30 for each day of completion. Participants completed questionnaires while in treatment and ceased symptom reporting when terminating treatment.

For patients completing questionnaires by Web they were prompted to do so weekly by receiving a notification in e‐Boks, a national communication system between authorities, companies, and citizens ensuring delivery of official communication regardless of geographical home address ensuring secure data delivery.^22^ A link and password to the questionnaire in the Ambuflex software was sent by notification in e‐Boks and reminders were sent one and two days after the weekly questionnaire.^19^

The specific scores from the EORTC and HADS questionnaires are hence the aim of this study reported elsewhere.^4^, ^23^

### 2.3 PRO‐CTCAE

The PRO‐CTCAE Item library consists of 78 symptom items explored by 128 questions as many symptoms are explored by attributes on frequency (F), severity (S), interference with daily activities (I) and/or presence (P).^24^ The scoring follows a Likert‐like scale 0‐4 with higher scores indicating worsening of the symptom. The only exception to this concept of the 45 chosen items was the item on ‘decreased libido’ in which the scoring is 0‐6; the score 5 with the wording ‘not sexually active’ and the score 6 with the wording ‘prefer not to answer’. For these response options within this item, the scoring was changed to ‘‘missing’’ in order to avoid overestimation of the burden of the decreased libido. For PRO‐CTCAE “frequency” items with a score of zero and blank attributing items on severity and/or interference with daily activities, the skipped items were assumed to be zero and were thus imputed.

### 2.4 Statistical analysis

To test the association between two instruments as a way of testing for construct validity when developing new measurement tools correlation analyses were performed. These analyses verify that the tool in question can distinguish between groups of patients with differences in the symptom under study. The analyses can also identify shifts in symptom severity within the same patient when using a well‐known tool as the anchor, in this case, the EORTC QLQ‐C30. Scatter plots were used to check for monotonicity, as a determination of strength and direction of the correlation in question, between the given PRO‐CTCAE item and calculated global QoL score, including all subdomains: physical function, role function, emotional function, cognitive function, and social function. The spearman's test was then performed to test for correlations between the PRO‐CTCAE item and QoL and its subdomains. Correlation analyses were performed for the total population and for the locally advanced and metastatic population separately. Absolute values of rho (r_s_, in either direction from 0) of 0‐0.19 were regarded as very weak, 0.2‐0.39 as weak, 0.40‐0.59 as moderate, 0.6‐0.79 as strong and 0.8‐1 as very strong correlation. A PRO‐CTCAE symptom was included in the above r_s_ categories if one or more correlation coefficient between a PRO‐CTCAE item and the expected domain was found within the given intervals. Descriptive analysis was used to describe the symptom burden from PRO‐CTCAE scores. Linear regression analysis was performed to estimate the relation between summarized PRO‐CTCAE scores and QoL and its subdomains.

## 3 RESULTS

**FIGURE 1:**
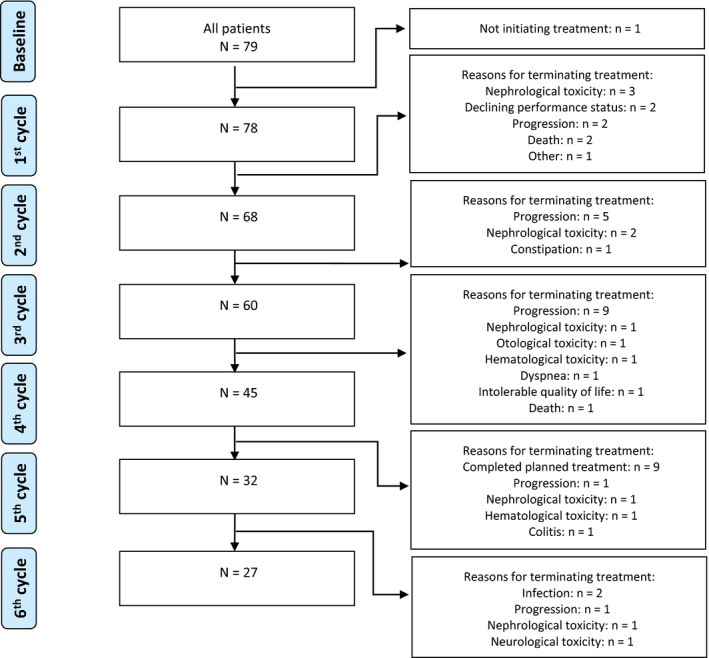
Consort diagram of the clinical course of patients in this study, N = 79. Explanations for treatment cessation is given in boxes on the right hand side, treatment cycles are illustrated on the left‐hand side

From August 2017 to September 2018, a total of 122 patients were screened for eligibility. In total 27 patients (22%) did not fulfill inclusion criteria due to either poor performance status inhibiting treatment initiation (n = 14, 11%), enrollment into a clinical trial (n = 2, 2%), initiating radiotherapy instead of chemo‐/immunotherapy (n = 1, 1%), synchronic metastatic cancer of another site (n = 2, 2%) or lack of access to electronic communication with authorities through e‐Boks™ (n = 8, 7%). A further three patients (2%) were missed at treatment initiation and three (2%) declined treatment altogether. Participation was declined by 10/122 patients (8%) leaving 79 patients who completed written informed consent, one of whom did not initiate questionnaire completion due to a cerebral stroke on the day of completing informed consent. The clinical data of the participating patients are listed in Table 1. For all participants, the median age was 68 (range 35‐82) and the majority of patients were treated for metastatic disease (67%). Figure 1 displays the clinical course of the patients throughout treatment.

**TABLE 1:** Patient characteristics from study 1 and 2

Clinical data		Total n = 79 (%)	Study 1 N = 30 (%)	Study 2 n = 49 (%)
Gender	Men	64 (81)	22 (73)	42 (86)
	Women	15 (19)	8 (27)	7 (14)
Median age, yrs. (range)		68 (35‐82)	68 (35‐82)	68(48‐80)
Stage	Locally advanced	26 (33)	13 (43)	13 (27)
	Metastatic	53 (67)	17 (57)	36 (73)
Treatment^a^	Cisplatin + gemcitabine	46 (59)	22 (76)	24 (49)
	Carboplatin + gemcitabine	9 (11)	6 (21)	3 (6)
	Vinflunine	3 (4)	1 (3)	2 (4)
	Pembrolizumab	20 (26)	0 (0)	20 (41)

^a^ One patient in study 1 never started treatment due to cerebral stroke before initiation of treatment.

A total of 711 questionnaires were completed by the participating 78 patients. The median number of completed questionnaires was 9 (range 0‐20). The overall completion rate was 76%. Twenty patients completed questionnaires on paper and 58 by web‐browser in Ambuflex. Analysis of missing data revealed an overall completion of 93% and missing data in 7% of the 724 questionnaires, all with 158 items equaling 8007 missing item responses out of a total of 114.392 item responses. After imputing zeros in the PRO‐CTCAE items following a zero in the frequency item, the missing data analysis showed 6% missing data. The transformation of values 5 and 6 to ‘missing’ for the item ‘decreased libido’ was not included as missing data in this analysis as they were completed by the patients.

When analyzing the relationship between all single PRO‐CTCAE items and QLQ‐C30 quality of life including subdomains (see Table S1), positive correlations with the expected direction of the correlation were found on almost all items. The items with the highest correlations with QoL were the PRO‐CTCAE items anxiety (F) and emotional function (*r*_s_ = −0.603, *P* < .0001), concentration (S), and cognitive function (*r*_s_ = −0.704, *P* < .0001), discouraged (F) and emotional function (*r*_s_ = −0.659, *P* < .0001), fatigue (S) and role function (*r*_s_ = −0.659, *P* < .0001), memory (S) and cognitive function (*r*_s_ = −0.784, *P* < .0001), and sad (F) and emotional function (*r*_s_ = −0.711, *P* < .0001), all with the expected direction of correlation with QoL, see Table 2 and Table S1. No items reached the predefined threshold for very strong correlations (*r*_s_ > 0.8). The PRO‐CTCAE items concerning abdominal pain, dizziness, memory, muscle pain, nausea, pain, and shortness of breath all reached moderate correlations of Spearman's rho 0.4‐0.59. The items with the lowest rank‐order correlations with the expected QoL domain were the PRO‐CTCAE items concerning pain and swelling at the injection site (P) (Physical function: *r*_s_ = 0.002, *P* = .958), urinary frequency (F) (Physical function: *r*_s_ = 0.047, *P* = .231), urinary incontinence (F) (Physical function: *r*_s_ = −0.066, *P* = .091), and urinary urgency (F) (Physical function: *r*_s_ = −0.002, *P* = .966), all with very weak correlations, nonsignificant and with converging directions of the correlations. For the corresponding interference with daily activities items (I) of the urinary items, the correlations were very weak or weak but significant within the expected domain of QoL. No significant differences were found between the patients with locally advanced disease vs. metastatic disease although patients with locally advanced disease generally experienced higher correlations between a larger amount of symptoms (more symptoms with weak *r*_s_) and QoL than the metastatic population (more symptoms with very weak *r*_s_).

**TABLE 2:** Summarized Spearman's rank‐order correlations between PRO‐CTCAE items and EORTC QLQ‐C30 domains

All patients N = 78				
Very strong (0.8‐1)	Strong (0.6‐0.79)	Moderate (0.4‐0.59)	Weak (0.2‐0.39)	Very weak (0‐0.19)
	Anxiety F (−0.603)	Abdominal pain I (−0.481)	Blurred vision S (−0.320)	Change in urine colour P (−0.197)
	Concentration S (−0.704)	Decreased appetite I (−0.408)	Chills S (−0.205)	Constipation S (−0.079)
	Discouraged F (−0.659)	Dizziness S (−0.496)	Decreased libido S (−0.280)	Cough S (−0.110)
	Fatigue S (−0.659)	Muscle pain I (−0.459)	Difficulty swallowing S (−0.270)	Diarrhea F (−0.133)
	Memory S (−0.784)	Nausea F (−0.412)	Dry mouth S (−0.386)	Hair loss A (−0.144)
	Sad F (−0.711)	Pain I (−0.512)	Dry skin S (−0.251)	Headache I (−0.169)
		Shortness of breath I (−0.572)	Heart palpitations F (−0.266)	Heartburn F (−0.197)
			Hives P (−0.267)	Itching S (−0.190)
			Hot flashes F (−0.236)	Mouth/throat sores I (−0.170)
			Increased sweating F (−0.219)	Pain and swelling at injection site P (−0.004)
			Insomnia I (−0.321)	Painful urination S (−0.169)
			Joint pain I (−0.363)	Ringing in ears S (−0.108)
			Numbness & tingling I (−0.286)	Urinary frequency I (−0.131)
			Rash P (−0.283)	Urinary incontinence F (−0.053)
			Swelling of arms or legs I (−0.274)	Urinary urgency I (−0.091)
			Taste changes S (−0.275)	
			Vomiting F (−0.234)	
**Patients with locally advanced disease N = 26**				
	Anxiety F (−0.642)	Abdominal pain I (−0.456)	Blurred vision I (−0.301)	Cough S (−0.110)
	Concentration S (−0.715)	Decreased appetite S (−0.500)	Change in urine color P (−0.357)	Diarrhea F (−0.155)
	Discouraged F (−0.605)	Dizziness I (−0.483)	Chills S (−0.232)	Itching S (−0.158)
	Memory S (−0.758)	Dry mouth S (−0.429)	Constipation S (−0.344)	Mouth/throat sores I (−0.097)
	Sad F (−0.621)	Fatigue I (−0.557)	Decreased libido S (−0.200)	Numbness & tingling I (−0.183)
		Heart palpitations S (−0.404)	Difficulty swallowing S (−0.329)	Pain and swelling at injection site P (−0.096)
		Nausea F (−0.545)	Dry skin S (−0.256)	Painful urination S (0.106)
		Pain I (−0.514)	Hair loss A (−0.278)	Urinary frequency I (−0.178)
		Shortness of breath I (−0.528)	Headache I (−0.382)	Urinary incontinence F (0.088)
		Taste changes S (−0.487)	Heartburn F (−0.430)	Urinary urgency I (−0.177)
			Hives P (−0.267)	Vomiting F (−0.180)
			Hot flashes F (−0.242)	
			Increased sweating F (−0.257)	
			Insomnia I (−0.335)	
			Joint pain I (−0.354)	
			Muscle pain I (−0.419)	
			Rash P (−0.218)	
			Ringing in ears S (−0.322)	
			Swelling of arms or legs S (−0.206)	
Patients with metastatic disease N = 52				
	Concentration S (−0.707)	Abdominal pain I (−0.483)	Blurred vision S (−0.369)	Constipation S (−0.082)
	Discouraged F (−0.678)	Anxiety F (−0.577)	Change in urine color P (−0.339)	Cough S (−0.110)
	Fatigue S (−0.695)	Decreased appetite S (−0.404)	Chills S (−0.233)	Hair loss A (−0.110)
	Memory S (−0.799)	Decreased libido S (−0.443)	Diarrhea F (−0.283)	Headache S (−0.086)
	Sad F (−0.751)	Dizziness S (−0.547)	Difficulty swallowing S (−0.312)	Hives P (−0.194)
		Dry mouth S (−0.415)	Dry skin S (−0.305)	Painful urination S (0.108)
		Pain F (−0.512)	Heartburn F (−0.245)	Ringing in ears S (−0.057)
		Shortness of breath I (−0.575)	Heart palpitations S (−0.236)	Urinary frequency I (−0.178)
			Hot flashes F (−0.269)	Urinary incontinence I (−0.128)
			Increased sweating F (−0.236)	Urinary urgency I (−0.113)
			Insomnia I (−0.361)	
			Itching S (−0.259)	
			Joint pain I (−0.265)	
			Mouth/throat sores S (−0.295)	
			Muscle pain S (−0.409)	
			Nausea F (−0.364)	
			Numbness & tingling I (−0.288)	
			Pain and swelling at injection site P (−0.214)	
			Rash P (−0.292)	
			Swelling of arms or legs S (−0.293)	
			Taste changes S (−0.210)	
			Vomiting F (−0.276)	

Note

PRO‐CTCAE items were grouped in the above groups very weak, weak, moderate, strong, very strong if one or more item corresponded accordingly to the expected quality of life domain. Values in (parentheses): Rs correlation coefficient. F: frequency, S: severity, I: interference. Green color indicates agreement across all groups.

**FIGURE 2:**
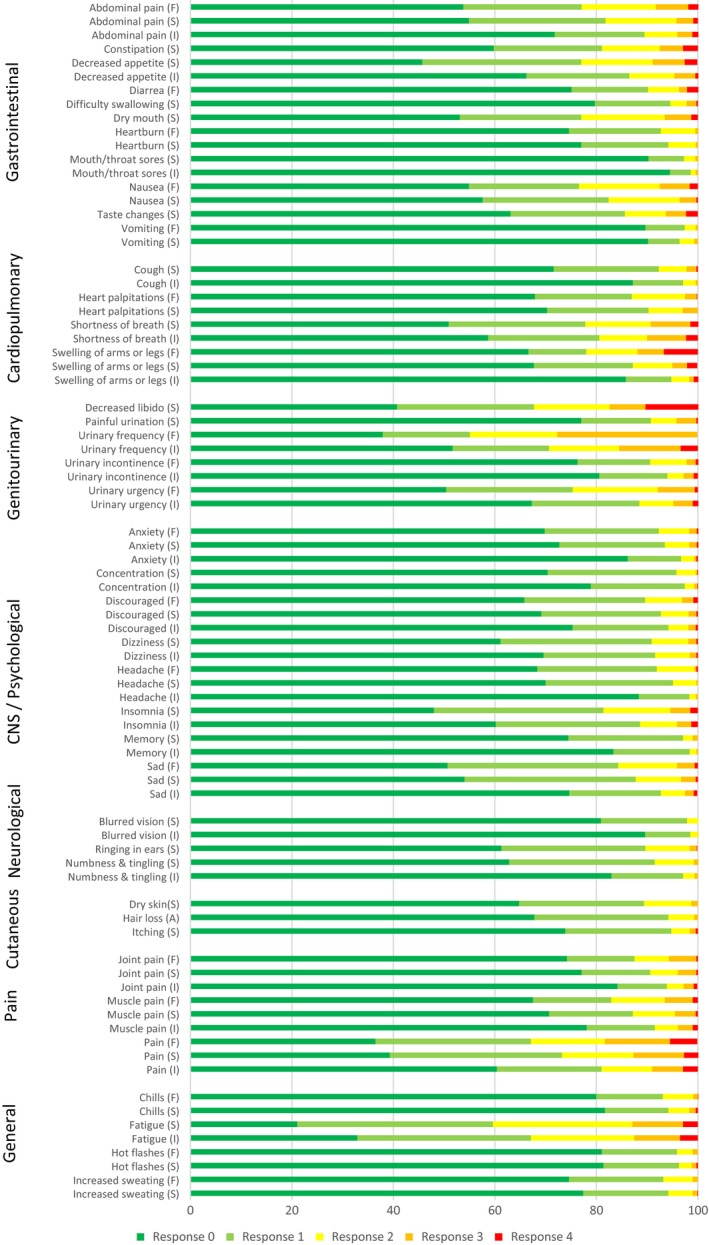
Distribution of PRO‐CTCAE responses for all patients during treatment. Item legends: F: Frequency, S: Severity, I: Interference with daily activities, A: Amount. Items attributed by Presence (P) (yes/no) are not included in this figure. Response options vary depending on item attribute: Frequency: 0 = ‘Never’, 1 = ‘Rarely’, 2 = ‘Occasionally’, 3 = ‘Frequently’, 4 = ‘Almost constantly’. Severity: 0 = ‘None’, 1 = ‘Mild’, 2 = ‘Moderate’, 3 = ‘Severe’, 4 = ‘Very severe’. Interference: 0 = ‘Not at all’, 1 = ‘A little bit’, 2 = ‘Somewhat’, 3 = ‘Quite a bit’, 4 = ‘Very much’. Amount: 0 = ‘Not at all’, 1 = ‘A little bit’, 2 = ‘Somewhat’, 3 = ‘Quite a bit’, 4 = ‘Very much’

Figure 2 displays the total symptom burden by PRO‐CTCAE scores during treatment. Linear regression analysis showed significant correlations between all QoL domains and summarized PRO‐CTCAE scores (*P* < .0001 for all analyses, see Table 3). When performing the same analyses without inclusion of the psychological items with strong correlations from Table 2, the estimates did not change significantly.

**TABLE 3:** Regression analysis between summarized PRO‐CTCAE score and quality of life domains

Summarized PRO‐CTCAE score	EORTC QLQ‐C30 domain	Coefficient	95% Confidence interval	
			Lower	Upper
	Global QoL	−0.452*	−0.494	−0.411
	Physical Function	−0.400*	−0.445	−0.356
	Role Function	−0.573*	−0.637	−0.506
	Emotional function	−0.303*	−0.341	−0.264
	Cognitive function	−0.310*	−0.345	−0.275
	Social Function	−0.404*	−0.453	‐0.356

Abbreviation: QoL, Quality of life.

* All coefficients statistically significant, *P* < .001.

## 4 DISCUSSION

This study identifies imperative symptoms for bladder cancer patients during chemo‐ or immunotherapy most likely to influence QoL. Interestingly the symptoms with the highest correlations are predominantly of a psychological nature whereas urinary symptoms known to be frequent for this population and over time anticipated to influence QoL the most, do not show positive or significant correlations with QoL. We find that our data may inform caregivers to focus on supportive care in these areas during treatment in order to best assist bladder cancer patients to achieve a better QoL. Likewise, this study may inform future investigators of imperative symptoms to report from a clinical trial for a comprehensive collection of the full symptom burden of enrolled patients for more transparent toxicity reporting in clinical trials.

Our findings of the correlations between the PRO‐CTCAE and the EORTC QLQ‐C30 are similar to that of Dueck et al in the validation process of the PRO‐CTCAE instrument in 2015.^18^ Interestingly, although the Dueck study population consisted of a mixed population of prostate and bladder cancer patients Dueck et al did not find a positive correlation, as in the present study, between urinary symptoms and QoL domains. For bladder cancer patients, urinary symptoms have long been the most frequently reported symptoms from clinical trials 25, 26 presumably because these symptoms are frequent in this population, as shown in Figure 2, but likely also because investigators assume that these symptoms are important for bladder cancer patients and reflect QoL. An explanation for the coherent findings of our study and the Dueck study may be that urological patients are well informed about urinary symptoms and are expecting the worst and thus are less affected by these symptoms. Our results demonstrate that a simple report of QoL and subdomains does not elicit the full picture of symptomatology relevant to the bladder cancer population. This is especially interesting for this population, as the first line treatment of choice is based on global QoL data from a phase 3 trial^14^ thus questioning the usability of such when not assimilating the full picture of symptomatology. Meanwhile, although urinary symptoms seem to have little influence on QoL, different aspects of urinary symptoms need to be handled by clinical staff whether it is pollakiuria, hematuria, urge or incontinence as these symptoms could indicate underlying infection. Conversely, psychological symptoms are in this study found to have high correlations with QoL and thus indicate a significant impact of these symptoms on QoL, perhaps as a result of sparse focus on these symptoms from a clinical perspective thus leaving the patients less prepared. The strong correlation between psychological items and QoL is, however, not unique to the bladder cancer population and is also seen in the Dueck study and for noncancer populations.^18^, ^27^, ^28^, ^29^ Our results therefore advocate for a fuller and more transparent reporting from clinical trials or daily clinical practice with a focus not only on QoL but also on selected PROs or vice versa. An approach as such would abrogate inconclusive reports of symptomatology from clinical trials and more meaningfully portray the patient's voice.

The symptoms with moderate or strong correlations are all general symptoms likely to reflect symptomology of a broad group of cancer patients and not specific to bladder cancer patients or to the stage of disease. With the added attention toward active management of PROs during treatment, this finding speaks for a general, nondisease specific, model of PROs during treatment if the aim is to reflect QoL. This approach has also been shown to be beneficial for patients in previous trials testing the impact of PROs.^6^, ^8^, ^9^ However, as supported above, some symptoms unique to the patient group at hand may be missed by using general PROs which could lead to unnecessary worsening of symptoms.

We found significant associations between summarized PRO‐CTCAE scores and QoL, including subdomains, in our linear regression model. When performing the same analysis without the inclusion of the psychological items found to have strong correlations from Table 2, the estimates did not change considerably. This finding indicates that the nonpsychological total symptom burden correlates with QoL and subdomains despite moderate or weak correlations in the Spearman's analyses as also reported in a previous study examining correlations between PRO‐CTCAE and QoL.^30^ This finding speaks into the concept above of a general PRO item model for active management of burdensome symptoms during treatment.

The strengths of this study include the prospective collection of data in a broad cohort of bladder cancer patients receiving active oncological treatment. This study is, to the best of our knowledge, the first to perform these analyses in a population of patients receiving chemo‐ or immunotherapy for advanced disease. We believe that the data are representative and that the results are applicable for a wide spectrum of bladder cancer patients. Also, with the disease‐specific PRO‐CTCAE item selection performed prior to our analysis and described in detail in a separate publication we believe that we in this study capture PROs relevant for the bladder cancer patients without adding substantial burden of questionnaire completion for the patients while reporting.

Some limitations do however need to be addressed. First, although correlation analysis from 724 questionnaires have been presented, only 78 patients report in this study thereby including a single patient's reports over time multiple times in the analysis. Patients responding to treatment and thus reporting for a longer period will therefore weigh more in this analysis potentially skewing the data and underestimating symptom burden in Figure 2. Second, while the completion rate of questionnaires was high in this study and our results should reflect a broad population of patients, this study would have benefitted from a supplementary qualitative research study with patient and physician interviews to be able to confirm our findings as correlation analyses cannot stand alone in presenting which symptoms affect QoL.

With the increasing focus on PROs as part of clinical trials investigators should be aware of the pitfalls of item selection when planning trials and especially of the items imperative for the population at question. Bladder cancer patients with advanced disease have a poor prognosis, partly due to a burden of comorbidity troubling treatment adherence.^4^, ^31^ Patients would therefore benefit from additional focus on essential PROs and QoL as nontoxic means of support during treatment. This study could inform investigators planning new trials and caregivers in daily clinical practice of a more comprehensive and transparent reporting of the full symptom burden during oncological treatment for bladder cancer patients.

## 5 Ethical approval and informed consent

The Danish National Data Protection Agency approved the conduction of studies included in this manuscript. The studies described in the manuscript were carried out prospectively during treatment for all patients completing written informed consent. In Denmark, the Danish Health Authority waivers the need for ethical approval for scientific studies assembling questionnaire data only. All procedures performed in studies involving human participants were in accordance with the ethical standards of the institution and the national research committee, the Danish Health Authority, and also with the 1964 Helsinki declaration and its later amendments or comparable ethical standards.

## Supporting information

Supplementary Material

## Data Availability

The data that support the findings of this study are available from Gry Assam Taarnhøj but restrictions apply to the availability of these data which were used under license for the current study, and so are not publicly available.
