# Supplementary material for: Patient reported symptoms associated with quality of life during chemo‐ or immunotherapy for bladder cancer patients with advanced disease
Source: Cancer Med. 2020 Mar 10;9(9):3078–87. doi: 10.1002/cam4.2958 (PMC7196058; doi:10.1002/cam4.2958)
Supplement: Supplementary file 1 — Supplementary Material [file CAM4-9-3078-s001.docx]

**Supplementary table A. Spearman’s correlation test between PRO-CTCAE items and quality of life domains.**

| **PRO-CTCAE Item** | **EORTC QLQ-C30 domain** | **All patients** | | | **Locally advanced disease** | | | **Metastatic disease** | | |
| --- | --- | --- | --- | --- | --- | --- | --- | --- | --- | --- |
|  |  | **N** | **R_s_** | ***P*-value** | **N** | **R_s_** | ***P*-value** | **N** | **R_s_** | ***P*-value** |
| Abdominal pain, F† | Global QoL‡ | 688 | -0.424 | <0.0001 | 276 | -0.332 | <0.0001 | 412 | -0.453 | <0.0001 |
|  | Physical Function | 683 | -0.325 | <0.0001 | 275 | -0.395 | <0.0001 | 408 | -0.283 | <0.0001 |
|  | Role Function | 688 | -0.373 | <0.0001 | 277 | -0.296 | <0.0001 | 411 | -0.398 | <0.0001 |
|  | Emotional function | 688 | -0.391 | <0.0001 | 276 | -0.331 | <0.0001 | 412 | -0.422 | <0.0001 |
|  | Cognitive function | 690 | -0.301 | <0.0001 | 277 | -0.282 | <0.0001 | 413 | -0.305 | <0.0001 |
|  | Social Function | 675 | -0.440 | <0.0001 | 262 | -0.370 | <0.0001 | 413 | -0.462 | <0.0001 |
| Abdominal pain, S† | Global QoL‡ | 688 | -0.388 | <0.0001 | 276 | -0.324 | <0.0001 | 412 | -0.397 | <0.0001 |
|  | Physical Function | 683 | -0.308 | <0.0001 | 275 | -0.364 | <0.0001 | 408 | -0.278 | <0.0001 |
|  | Role Function | 688 | -0.353 | <0.0001 | 277 | -0.277 | <0.0001 | 411 | -0.384 | <0.0001 |
|  | Emotional function | 688 | -0.352 | <0.0001 | 276 | -0.325 | <0.0001 | 412 | -0.359 | <0.0001 |
|  | Cognitive function | 690 | -0.260 | <0.0001 | 277 | -0.257 | <0.0001 | 413 | -0.248 | <0.0001 |
|  | Social Function | 675 | -0.415 | <0.0001 | 262 | -0.356 | <0.0001 | 413 | -0.433 | <0.0001 |
| Abdominal pain, I† | Global QoL‡ | 687 | -0.448 | <0.0001 | 276 | -0.447 | <0.0001 | 411 | -0.417 | <0.0001 |
|  | Physical Function | 682 | -0.377 | <0.0001 | 275 | -0.456 | <0.0001 | 407 | -0.309 | <0.0001 |
|  | Role Function | 687 | -0.419 | <0.0001 | 277 | -0.338 | <0.0001 | 410 | -0.442 | <0.0001 |
|  | Emotional function | 687 | -0.368 | <0.0001 | 276 | -0.426 | <0.0001 | 411 | -0.333 | <0.0001 |
|  | Cognitive function | 689 | -0.344 | <0.0001 | 277 | -0.437 | <0.0001 | 412 | -0.284 | <0.0001 |
|  | Social Function | 674 | -0.481 | <0.0001 | 262 | -0.438 | <0.0001 | 412 | -0.483 | <0.0001 |
| Anxiety, F† | Global QoL‡ | 684 | -0.365 | <0.0001 | 273 | -0.487 | <0.0001 | 411 | -0.296 | <0.0001 |
|  | Physical Function | 679 | -0.213 | <0.0001 | 272 | -0.400 | <0.0001 | 407 | -0.108 | 0.029 |
|  | Role Function | 684 | -0.249 | <0.0001 | 274 | -0.298 | <0.0001 | 410 | -0.227 | <0.0001 |
|  | Emotional function | 684 | -0.603 | <0.0001 | 273 | -0.642 | <0.0001 | 411 | -0.577 | <0.0001 |
|  | Cognitive function | 686 | -0.222 | <0.0001 | 274 | -0.415 | <0.0001 | 412 | -0.093 | 0.060 |
|  | Social Function | 671 | -0.340 | <0.0001 | 259 | -0.383 | <0.0001 | 412 | -0.325 | <0.0001 |
| Anxiety, S† | Global QoL‡ | 684 | -0.330 | <0.0001 | 273 | -0.414 | <0.0001 | 411 | -0.276 | <0.0001 |
|  | Physical Function | 679 | -0.195 | <0.0001 | 272 | -0.324 | <0.0001 | 407 | -0.115 | 0.020 |
|  | Role Function | 684 | -0.249 | <0.0001 | 274 | -0.275 | <0.0001 | 410 | -0.233 | <0.0001 |
|  | Emotional function | 684 | -0.572 | <0.0001 | 273 | -0.588 | <0.0001 | 411 | -0.557 | <0.0001 |
|  | Cognitive function | 686 | -0.186 | <0.0001 | 274 | -0.320 | <0.0001 | 412 | -0.094 | 0.058 |
|  | Social Function | 671 | -0.291 | <0.0001 | 259 | -0.279 | <0.0001 | 412 | -0.302 | <0.0001 |
| Anxiety, I† | Global QoL‡ | 683 | -0.316 | <0.0001 | 273 | -0.461 | <0.0001 | 410 | -0.237 | <0.0001 |
|  | Physical Function | 678 | -0.217 | <0.0001 | 272 | -0.463 | <0.0001 | 406 | -0.059 | 0.235 |
|  | Role Function | 683 | -0.247 | <0.0001 | 274 | -0.349 | <0.0001 | 409 | -0.191 | <0.0001 |
|  | Emotional function | 683 | -0.467 | <0.0001 | 273 | -0.564 | <0.0001 | 410 | -0.404 | <0.0001 |
|  | Cognitive function | 685 | -0.176 | <0.0001 | 274 | -0.372 | <0.0001 | 411 | -0.041 | 0.404 |
|  | Social Function | 670 | -0.261 | <0.0001 | 259 | -0.406 | <0.0001 | 411 | -0.184 | <0.0001 |
| Blurred vision, S† | Global QoL‡ | 686 | -0.236 | <0.0001 | 275 | -0.205 | 0.001 | 411 | -0.215 | <0.0001 |
|  | Physical Function | 681 | -0.320 | <0.0001 | 274 | -0.198 | 0.001 | 407 | -0.369 | <0.0001 |
|  | Role Function | 686 | -0.281 | <0.0001 | 276 | -0.287 | <0.0001 | 410 | -0.257 | <0.0001 |
|  | Emotional function | 686 | -0.192 | <0.0001 | 275 | -0.262 | <0.0001 | 411 | -0.153 | 0.002 |
|  | Cognitive function | 688 | -0.298 | <0.0001 | 276 | -0.166 | 0.006 | 412 | -0.362 | <0.0001 |
|  | Social Function | 673 | -0.233 | <0.0001 | 261 | -0.068 | 0.276 | 412 | -0.305 | <0.0001 |
| Blurred vision, I† | Global QoL‡ | 685 | -0.234 | <0.0001 | 275 | -0.268 | <0.0001 | 410 | -0.179 | <0.0001 |
|  | Physical Function | 680 | -0.276 | <0.0001 | 274 | -0.278 | <0.0001 | 406 | -0.248 | <0.0001 |
|  | Role Function | 685 | -0.224 | <0.0001 | 276 | -0.301 | <0.0001 | 409 | -0.164 | 0.001 |
|  | Emotional function | 685 | -0.143 | <0.0001 | 275 | -0.251 | <0.0001 | 410 | -0.094 | 0.056 |
|  | Cognitive function | 687 | -0.208 | <0.0001 | 276 | -0.214 | <0.0001 | 411 | -0.195 | <0.0001 |
|  | Social Function | 672 | -0.169 | <0.0001 | 261 | -0.171 | 0.006 | 411 | -0.148 | 0.003 |
| Change in usual urine colour, P† | Global QoL‡ | 680 | -0.093 | 0.016 | 271 | -0.271 | <0.0001 | 409 | 0.115 | 0.020 |
|  | Physical Function | 675 | -0.061 | 0.114 | 270 | -0.357 | <0.0001 | 405 | 0.339 | <0.0001 |
|  | Role Function | 680 | -0.076 | 0.049 | 272 | -0.308 | <0.0001 | 408 | 0.183 | <0.0001 |
|  | Emotional function | 680 | -0.129 | 0.001 | 271 | -0.332 | <0.0001 | 409 | 0.035 | 0.486 |
|  | Cognitive function | 682 | -0.115 | 0.003 | 272 | -0.290 | <0.0001 | 410 | 0.063 | 0.202 |
|  | Social Function | 668 | -0.197 | <0.0001 | 258 | -0.361 | <0.0001 | 410 | -0.010 | 0.844 |
| Chills, F† | Global QoL‡ | 685 | -0.190 | <0.0001 | 274 | -0.156 | 0.010 | 411 | -0.221 | <0.0001 |
|  | Physical Function | 680 | -0.174 | <0.0001 | 273 | -0.200 | 0.001 | 407 | -0.212 | <0.0001 |
|  | Role Function | 685 | -0.163 | <0.0001 | 275 | -0.138 | 0.022 | 410 | -0.194 | <0.0001 |
|  | Emotional function | 685 | -0.135 | <0.0001 | 274 | -0.116 | 0.056 | 411 | -0.151 | 0.002 |
|  | Cognitive function | 687 | -0.164 | <0.0001 | 275 | -0.099 | 0.103 | 412 | -0.209 | <0.0001 |
|  | Social Function | 672 | -0.132 | 0.001 | 260 | -0.118 | 0.057 | 412 | -0.150 | 0.002 |
| Chills, S† | Global QoL‡ | 685 | -0.205 | <0.0001 | 274 | -0.193 | 0.001 | 411 | -0.215 | <0.0001 |
|  | Physical Function | 680 | -0.205 | <0.0001 | 273 | -0.232 | <0.0001 | 407 | -0.231 | <0.0001 |
|  | Role Function | 685 | -0.184 | <0.0001 | 275 | -0.185 | 0.002 | 410 | -0.193 | <0.0001 |
|  | Emotional function | 685 | -0.149 | <0.0001 | 274 | -0.144 | 0.017 | 411 | -0.156 | 0.002 |
|  | Cognitive function | 687 | -0.198 | <0.0001 | 275 | -0.138 | 0.023 | 412 | -0.233 | <0.0001 |
|  | Social Function | 672 | -0.168 | <0.0001 | 260 | -0.184 | 0.003 | 412 | -0.165 | 0.001 |
| Concentration, S† | Global QoL‡ | 685 | -0.398 | <0.0001 | 274 | -0.462 | <0.0001 | 411 | -0.363 | <0.0001 |
|  | Physical Function | 680 | -0.374 | <0.0001 | 273 | -0.421 | <0.0001 | 407 | -0.389 | <0.0001 |
|  | Role Function | 685 | -0.303 | <0.0001 | 275 | -0.325 | <0.0001 | 410 | -0.303 | <0.0001 |
|  | Emotional function | 685 | -0.410 | <0.0001 | 274 | -0.576 | <0.0001 | 411 | -0.313 | <0.0001 |
|  | Cognitive function | 687 | -0.704 | <0.0001 | 275 | -0.715 | <0.0001 | 412 | -0.707 | <0.0001 |
|  | Social Function | 672 | -0.345 | <0.0001 | 260 | -0.345 | <0.0001 | 412 | -0.365 | <0.0001 |
| Concentration, I† | Global QoL‡ | 684 | -0.392 | <0.0001 | 274 | -0.457 | <0.0001 | 410 | -0.356 | <0.0001 |
|  | Physical Function | 678 | -0.327 | <0.0001 | 273 | -0.406 | <0.0001 | 405 | -0.306 | <0.0001 |
|  | Role Function | 683 | -0.244 | <0.0001 | 275 | -0.258 | <0.0001 | 408 | -0.247 | <0.0001 |
|  | Emotional function | 684 | -0.346 | <0.0001 | 274 | -0.513 | <0.0001 | 410 | -0.248 | <0.0001 |
|  | Cognitive function | 685 | -0.621 | <0.0001 | 275 | -0.674 | <0.0001 | 410 | -0.594 | <0.0001 |
|  | Social Function | 670 | -0.301 | <0.0001 | 260 | -0.376 | <0.0001 | 410 | -0.269 | <0.0001 |
| Constipation, S† | Global QoL‡ | 687 | -0.192 | <0.0001 | 276 | -0.285 | <0.0001 | 411 | -0.201 | <0.0001 |
|  | Physical Function | 683 | -0.079 | 0.039 | 275 | -0.291 | <0.0001 | 408 | -0.082 | 0.097 |
|  | Role Function | 687 | -0.127 | 0.001 | 277 | -0.229 | <0.0001 | 410 | -0.133 | 0.007 |
|  | Emotional function | 687 | -0.213 | <0.0001 | 276 | -0.214 | <0.0001 | 411 | -0.233 | <0.0001 |
|  | Cognitive function | 689 | -0.118 | 0.002 | 277 | -0.268 | <0.0001 | 412 | -0.049 | 0.320 |
|  | Social Function | 674 | -0.189 | <0.0001 | 262 | -0.344 | <0.0001 | 412 | -0.139 | 0.005 |
| Cough, S† | Global QoL‡ | 687 | -0.141 | <0.0001 | 276 | -0.165 | 0.006 | 411 | -0.180 | <0.0001 |
|  | Physical Function | 683 | -0.110 | 0.004 | 275 | -0.196 | 0.001 | 408 | -0.152 | 0.002 |
|  | Role Function | 687 | -0.045 | 0.240 | 277 | -0.128 | 0.034 | 410 | -0.038 | 0.448 |
|  | Emotional function | 687 | -0.207 | <0.0001 | 276 | -0.299 | <0.0001 | 411 | -0.167 | 0.001 |
|  | Cognitive function | 689 | -0.232 | <0.0001 | 277 | -0.279 | <0.0001 | 412 | -0.237 | <0.0001 |
|  | Social Function | 674 | -0.098 | 0.011 | 262 | -0.132 | 0.033 | 412 | -0.109 | 0.027 |
| Cough, I† | Global QoL‡ | 686 | -0.167 | <0.0001 | 276 | -0.249 | <0.0001 | 410 | -0.139 | 0.005 |
|  | Physical Function | 682 | -0.108 | 0.005 | 275 | -0.243 | <0.0001 | 407 | -0.077 | 0.120 |
|  | Role Function | 686 | 0.003 | 0.931 | 277 | -0.074 | 0.220 | 409 | 0.030 | 0.546 |
|  | Emotional function | 686 | -0.192 | <0.0001 | 276 | -0.309 | <0.0001 | 410 | -0.126 | 0.011 |
|  | Cognitive function | 688 | -0.203 | <0.0001 | 277 | -0.367 | <0.0001 | 411 | -0.100 | 0.043 |
|  | Social Function | 673 | -0.099 | 0.010 | 262 | -0.203 | 0.001 | 411 | -0.053 | 0.287 |
| Decreased appetite, S† | Global QoL‡ | 689 | -0.434 | <0.0001 | 275 | -0.478 | <0.0001 | 414 | -0.388 | <0.0001 |
|  | Physical Function | 684 | -0.347 | <0.0001 | 274 | -0.499 | <0.0001 | 410 | -0.270 | <0.0001 |
|  | Role Function | 689 | -0.439 | <0.0001 | 276 | -0.500 | <0.0001 | 413 | -0.404 | <0.0001 |
|  | Emotional function | 689 | -0.339 | <0.0001 | 275 | -0.489 | <0.0001 | 414 | -0.245 | <0.0001 |
|  | Cognitive function | 691 | -0.252 | <0.0001 | 276 | -0.299 | <0.0001 | 415 | -0.206 | <0.0001 |
|  | Social Function | 676 | -0.369 | <0.0001 | 261 | -0.371 | <0.0001 | 415 | -0.355 | <0.0001 |
| Decreased appetite, I† | Global QoL‡ | 686 | -0.408 | <0.0001 | 275 | -0.463 | <0.0001 | 411 | -0.359 | <0.0001 |
|  | Physical Function | 680 | -0.297 | <0.0001 | 274 | -0.431 | <0.0001 | 406 | -0.217 | <0.0001 |
|  | Role Function | 685 | -0.373 | <0.0001 | 276 | -0.396 | <0.0001 | 409 | -0.358 | <0.0001 |
|  | Emotional function | 686 | -0.314 | <0.0001 | 275 | -0.436 | <0.0001 | 411 | -0.239 | <0.0001 |
|  | Cognitive function | 687 | -0.239 | <0.0001 | 276 | -0.392 | <0.0001 | 411 | -0.140 | 0.005 |
|  | Social Function | 672 | -0.306 | <0.0001 | 261 | -0.338 | <0.0001 | 411 | -0.279 | <0.0001 |
| Decreased libido, S† | Global QoL‡ | 240 | -0.190 | 0.003 | 92 | -0.008 | 0.942 | 148 | -0.253 | 0.002 |
|  | Physical Function | 238 | -0.163 | 0.012 | 90 | -0.144 | 0.177 | 148 | 0.145 | 0.078 |
|  | Role Function | 241 | -0.082 | 0.207 | 93 | -0.200 | 0.055 | 148 | 0.015 | 0.860 |
|  | Emotional function | 241 | -0.280 | <0.0001 | 93 | -0.179 | 0.086 | 148 | -0.325 | <0.0001 |
|  | Cognitive function | 241 | -0.233 | <0.0001 | 93 | 0.078 | 0.455 | 148 | -0.443 | <0.0001 |
|  | Social Function | 241 | -0.088 | 0.173 | 93 | -0.056 | 0.591 | 148 | -0.086 | 0.299 |
| Diarrea, F† | Global QoL‡ | 687 | -0.133 | <0.0001 | 275 | 0.155 | 0.010 | 412 | -0.283 | <0.0001 |
|  | Physical Function | 682 | -0.091 | 0.018 | 274 | 0.058 | 0.339 | 408 | -0.157 | 0.001 |
|  | Role Function | 687 | -0.120 | 0.002 | 276 | 0.132 | 0.029 | 411 | -0.238 | <0.0001 |
|  | Emotional function | 687 | -0.143 | <0.0001 | 275 | 0.137 | 0.023 | 412 | -0.291 | <0.0001 |
|  | Cognitive function | 689 | -0.048 | 0.207 | 276 | 0.087 | 0.150 | 413 | -0.108 | 0.028 |
|  | Social Function | 674 | -0.123 | 0.001 | 261 | 0.087 | 0.161 | 413 | -0.214 | <0.0001 |
| Difficulty swallowing, S† | Global QoL‡ | 687 | -0.225 | <0.0001 | 276 | -0.301 | <0.0001 | 411 | -0.189 | <0.0001 |
|  | Physical Function | 682 | -0.270 | <0.0001 | 275 | -0.329 | <0.0001 | 407 | -0.312 | <0.0001 |
|  | Role Function | 687 | -0.213 | <0.0001 | 277 | -0.234 | <0.0001 | 410 | -0.218 | <0.0001 |
|  | Emotional function | 687 | -0.183 | <0.0001 | 276 | -0.389 | <0.0001 | 411 | -0.068 | 0.171 |
|  | Cognitive function | 689 | -0.374 | <0.0001 | 277 | -0.292 | <0.0001 | 412 | -0.438 | <0.0001 |
|  | Social Function | 674 | -0.247 | <0.0001 | 262 | -0.298 | <0.0001 | 412 | -0.235 | <0.0001 |
| Discouraged, F† | Global QoL‡ | 684 | -0.557 | <0.0001 | 273 | -0.570 | <0.0001 | 411 | -0.522 | <0.0001 |
|  | Physical Function | 679 | -0.459 | <0.0001 | 272 | -0.609 | <0.0001 | 407 | -0.337 | <0.0001 |
|  | Role Function | 684 | -0.439 | <0.0001 | 274 | -0.478 | <0.0001 | 410 | -0.386 | <0.0001 |
|  | Emotional function | 684 | -0.659 | <0.0001 | 273 | -0.605 | <0.0001 | 411 | -0.678 | <0.0001 |
|  | Cognitive function | 686 | -0.389 | <0.0001 | 274 | -0.401 | <0.0001 | 412 | -0.369 | <0.0001 |
|  | Social Function | 671 | -0.478 | <0.0001 | 259 | -0.501 | <0.0001 | 412 | -0.449 | <0.0001 |
| Discouraged, S† | Global QoL‡ | 684 | -0.526 | <0.0001 | 273 | -0.534 | <0.0001 | 411 | -0.491 | <0.0001 |
|  | Physical Function | 679 | -0.437 | <0.0001 | 272 | -0.577 | <0.0001 | 407 | -0.317 | <0.0001 |
|  | Role Function | 684 | -0.420 | <0.0001 | 274 | -0.454 | <0.0001 | 410 | -0.369 | <0.0001 |
|  | Emotional function | 684 | -0.632 | <0.0001 | 273 | -0.596 | <0.0001 | 411 | -0.646 | <0.0001 |
|  | Cognitive function | 686 | -0.360 | <0.0001 | 274 | -0.319 | <0.0001 | 412 | -0.364 | <0.0001 |
|  | Social Function | 671 | -0.437 | <0.0001 | 259 | -0.475 | <0.0001 | 412 | -0.400 | <0.0001 |
| Discouraged, I† | Global QoL‡ | 684 | -0.504 | <0.0001 | 273 | -0.547 | <0.0001 | 411 | -0.452 | <0.0001 |
|  | Physical Function | 679 | -0.423 | <0.0001 | 272 | -0.539 | <0.0001 | 407 | -0.314 | <0.0001 |
|  | Role Function | 684 | -0.411 | <0.0001 | 274 | -0.426 | <0.0001 | 410 | -0.375 | <0.0001 |
|  | Emotional function | 684 | -0.555 | <0.0001 | 273 | -0.548 | <0.0001 | 411 | -0.547 | <0.0001 |
|  | Cognitive function | 686 | -0.349 | <0.0001 | 274 | -0.369 | <0.0001 | 412 | -0.324 | <0.0001 |
|  | Social Function | 671 | -0.415 | <0.0001 | 259 | -0.501 | <0.0001 | 412 | -0.357 | <0.0001 |
| Dizziness, S† | Global QoL‡ | 687 | -0.334 | <0.0001 | 275 | -0.438 | <0.0001 | 412 | -0.241 | <0.0001 |
|  | Physical Function | 682 | -0.401 | <0.0001 | 274 | -0.404 | <0.0001 | 408 | -0.384 | <0.0001 |
|  | Role Function | 687 | -0.330 | <0.0001 | 276 | -0.312 | <0.0001 | 411 | -0.322 | <0.0001 |
|  | Emotional function | 687 | -0.165 | <0.0001 | 275 | -0.335 | <0.0001 | 412 | -0.067 | 0.177 |
|  | Cognitive function | 689 | -0.496 | <0.0001 | 276 | -0.393 | <0.0001 | 413 | -0.547 | <0.0001 |
|  | Social Function | 674 | -0.283 | <0.0001 | 261 | -0.311 | <0.0001 | 413 | -0.259 | <0.0001 |
| Dizziness, I† | Global QoL‡ | 686 | -0.346 | <0.0001 | 275 | -0.506 | <0.0001 | 411 | -0.218 | <0.0001 |
|  | Physical Function | 681 | -0.432 | <0.0001 | 274 | -0.483 | <0.0001 | 407 | -0.375 | <0.0001 |
|  | Role Function | 686 | -0.335 | <0.0001 | 276 | -0.358 | <0.0001 | 410 | -0.296 | <0.0001 |
|  | Emotional function | 686 | -0.154 | <0.0001 | 275 | -0.367 | <0.0001 | 411 | -0.040 | 0.422 |
|  | Cognitive function | 688 | -0.491 | <0.0001 | 276 | -0.436 | <0.0001 | 412 | -0.512 | <0.0001 |
|  | Social Function | 673 | -0.295 | <0.0001 | 261 | -0.436 | <0.0001 | 412 | -0.209 | <0.0001 |
| Dry mouth, S† | Global QoL‡ | 691 | -0.386 | <0.0001 | 276 | -0.401 | <0.0001 | 415 | -0.382 | <0.0001 |
|  | Physical Function | 686 | -0.384 | <0.0001 | 275 | -0.429 | <0.0001 | 411 | -0.415 | <0.0001 |
|  | Role Function | 691 | -0.286 | <0.0001 | 277 | -0.169 | 0.005 | 414 | -0.365 | <0.0001 |
|  | Emotional function | 691 | -0.182 | <0.0001 | 276 | -0.416 | <0.0001 | 415 | -0.051 | 0.304 |
|  | Cognitive function | 693 | -0.401 | <0.0001 | 277 | -0.394 | <0.0001 | 416 | -0.411 | <0.0001 |
|  | Social Function | 678 | -0.338 | <0.0001 | 262 | -0.380 | <0.0001 | 416 | -0.326 | <0.0001 |
| Dry skin, S† | Global QoL‡ | 687 | -0.194 | <0.0001 | 275 | -0.175 | 0.004 | 412 | -0.203 | <0.0001 |
|  | Physical Function | 682 | -0.251 | <0.0001 | 274 | -0.256 | <0.0001 | 408 | -0.305 | <0.0001 |
|  | Role Function | 687 | -0.195 | <0.0001 | 276 | -0.155 | 0.010 | 411 | -0.220 | <0.0001 |
|  | Emotional function | 687 | -0.082 | 0.032 | 275 | -0.047 | 0.432 | 412 | -0.106 | 0.032 |
|  | Cognitive function | 689 | -0.231 | <0.0001 | 276 | -0.046 | 0.450 | 413 | -0.345 | <0.0001 |
|  | Social Function | 674 | -0.187 | <0.0001 | 261 | -0.074 | 0.235 | 413 | -0.263 | <0.0001 |
| Fatigue, S† | Global QoL‡ | 684 | -0.611 | <0.0001 | 273 | -0.476 | <0.0001 | 411 | -0.631 | <0.0001 |
|  | Physical Function | 679 | -0.627 | <0.0001 | 272 | -0.496 | <0.0001 | 407 | -0.644 | <0.0001 |
|  | Role Function | 684 | -0.659 | <0.0001 | 274 | -0.538 | <0.0001 | 410 | -0.695 | <0.0001 |
|  | Emotional function | 684 | -0.432 | <0.0001 | 273 | -0.481 | <0.0001 | 411 | -0.379 | <0.0001 |
|  | Cognitive function | 686 | -0.443 | <0.0001 | 274 | -0.350 | <0.0001 | 412 | -0.470 | <0.0001 |
|  | Social Function | 671 | -0.553 | <0.0001 | 259 | -0.487 | <0.0001 | 412 | -0.556 | <0.0001 |
| Fatigue, I† | Global QoL‡ | 683 | -0.632 | <0.0001 | 273 | -0.557 | <0.0001 | 410 | -0.625 | <0.0001 |
|  | Physical Function | 678 | -0.649 | <0.0001 | 272 | -0.486 | <0.0001 | 406 | -0.655 | <0.0001 |
|  | Role Function | 683 | -0.640 | <0.0001 | 274 | -0.495 | <0.0001 | 409 | -0.687 | <0.0001 |
|  | Emotional function | 683 | -0.431 | <0.0001 | 273 | -0.523 | <0.0001 | 410 | -0.370 | <0.0001 |
|  | Cognitive function | 685 | -0.455 | <0.0001 | 274 | -0.423 | <0.0001 | 411 | -0.458 | <0.0001 |
|  | Social Function | 670 | -0.539 | <0.0001 | 259 | -0.490 | <0.0001 | 411 | -0.536 | <0.0001 |
| Hair loss, A† | Global QoL‡ | 686 | -0.062 | 0.106 | 275 | -0.140 | 0.020 | 411 | -0.036 | 0.462 |
|  | Physical Function | 681 | -0.050 | 0.189 | 274 | -0.124 | 0.040 | 407 | -0.080 | 0.106 |
|  | Role Function | 686 | -0.052 | 0.171 | 276 | -0.108 | 0.074 | 410 | -0.045 | 0.368 |
|  | Emotional function | 686 | -0.095 | 0.012 | 275 | -0.216 | <0.0001 | 411 | -0.039 | 0.432 |
|  | Cognitive function | 688 | -0.144 | <0.0001 | 276 | -0.278 | <0.0001 | 412 | -0.082 | 0.096 |
|  | Social Function | 673 | -0.064 | 0.096 | 261 | -0.052 | 0.402 | 412 | -0.110 | 0.025 |
| Headache, F† | Global QoL‡ | 686 | -0.024 | 0.527 | 275 | -0.084 | 0.166 | 411 | -0.089 | 0.071 |
|  | Physical Function | 681 | 0.086 | 0.025 | 274 | -0.134 | 0.027 | 407 | 0.066 | 0.182 |
|  | Role Function | 686 | 0.057 | 0.136 | 276 | -0.105 | 0.082 | 410 | 0.082 | 0.099 |
|  | Emotional function | 686 | -0.088 | 0.022 | 275 | -0.160 | 0.008 | 411 | -0.081 | 0.099 |
|  | Cognitive function | 688 | -0.027 | 0.475 | 276 | -0.198 | 0.001 | 412 | 0.038 | 0.438 |
|  | Social Function | 673 | 0.058 | 0.136 | 261 | -0.055 | 0.377 | 412 | 0.064 | 0.194 |
| Headache, S† | Global QoL‡ | 685 | -0.031 | 0.422 | 274 | -0.076 | 0.207 | 411 | -0.086 | 0.081 |
|  | Physical Function | 680 | 0.075 | 0.052 | 273 | -0.134 | 0.027 | 407 | 0.061 | 0.217 |
|  | Role Function | 685 | 0.039 | 0.305 | 275 | -0.113 | 0.061 | 410 | 0.072 | 0.144 |
|  | Emotional function | 685 | -0.093 | 0.015 | 274 | -0.170 | 0.005 | 411 | -0.074 | 0.133 |
|  | Cognitive function | 687 | -0.038 | 0.318 | 275 | -0.180 | 0.003 | 412 | 0.017 | 0.733 |
|  | Social Function | 672 | 0.048 | 0.218 | 260 | -0.055 | 0.373 | 412 | 0.057 | 0.245 |
| Headache, I† | Global QoL‡ | 684 | -0.169 | <0.0001 | 274 | -0.382 | <0.0001 | 410 | -0.042 | 0.396 |
|  | Physical Function | 679 | -0.104 | 0.007 | 273 | -0.325 | <0.0001 | 406 | -0.003 | 0.947 |
|  | Role Function | 684 | -0.105 | 0.006 | 275 | -0.255 | <0.0001 | 409 | -0.028 | 0.572 |
|  | Emotional function | 684 | -0.167 | <0.0001 | 274 | -0.375 | <0.0001 | 410 | -0.025 | 0.612 |
|  | Cognitive function | 686 | -0.133 | <0.0001 | 275 | -0.356 | <0.0001 | 411 | 0.019 | 0.706 |
|  | Social Function | 671 | -0.066 | 0.087 | 260 | -0.259 | <0.0001 | 411 | 0.042 | 0.399 |
| Heartburn, F† | Global QoL‡ | 688 | -0.197 | <0.0001 | 276 | -0.235 | <0.0001 | 412 | -0.245 | <0.0001 |
|  | Physical Function | 683 | -0.141 | <0.0001 | 275 | -0.430 | <0.0001 | 408 | -0.085 | 0.088 |
|  | Role Function | 688 | -0.155 | <0.0001 | 277 | -0.360 | <0.0001 | 411 | -0.079 | 0.112 |
|  | Emotional function | 688 | -0.290 | <0.0001 | 276 | -0.493 | <0.0001 | 412 | -0.168 | 0.001 |
|  | Cognitive function | 690 | -0.240 | <0.0001 | 277 | -0.445 | <0.0001 | 413 | -0.124 | 0.012 |
|  | Social Function | 675 | -0.198 | <0.0001 | 262 | -0.240 | <0.0001 | 413 | -0.235 | <0.0001 |
| Heartburn, S† | Global QoL‡ | 687 | -0.189 | <0.0001 | 276 | -0.230 | <0.0001 | 411 | -0.223 | <0.0001 |
|  | Physical Function | 682 | -0.141 | <0.0001 | 275 | -0.404 | <0.0001 | 407 | -0.096 | 0.054 |
|  | Role Function | 687 | -0.145 | <0.0001 | 277 | -0.333 | <0.0001 | 410 | -0.080 | 0.107 |
|  | Emotional function | 687 | -0.287 | <0.0001 | 276 | -0.494 | <0.0001 | 411 | -0.161 | 0.001 |
|  | Cognitive function | 689 | -0.233 | <0.0001 | 277 | -0.412 | <0.0001 | 412 | -0.133 | 0.007 |
|  | Social Function | 674 | -0.196 | <0.0001 | 262 | -0.246 | <0.0001 | 412 | -0.219 | <0.0001 |
| Heart palpitations, F† | Global QoL‡ | 688 | -0.266 | <0.0001 | 276 | -0.364 | <0.0001 | 412 | -0.218 | <0.0001 |
|  | Physical Function | 683 | -0.229 | <0.0001 | 275 | -0.367 | <0.0001 | 408 | -0.218 | <0.0001 |
|  | Role Function | 688 | -0.254 | <0.0001 | 277 | -0.328 | <0.0001 | 411 | -0.234 | <0.0001 |
|  | Emotional function | 688 | -0.220 | <0.0001 | 276 | -0.312 | <0.0001 | 412 | -0.168 | 0.001 |
|  | Cognitive function | 690 | -0.175 | <0.0001 | 277 | -0.333 | <0.0001 | 413 | -0.069 | 0.163 |
|  | Social Function | 675 | -0.194 | <0.0001 | 262 | -0.334 | <0.0001 | 413 | -0.112 | 0.023 |
| Heart palpitations, S† | Global QoL‡ | 687 | -0.263 | <0.0001 | 276 | -0.404 | <0.0001 | 411 | -0.187 | <0.0001 |
|  | Physical Function | 682 | -0.234 | <0.0001 | 275 | -0.375 | <0.0001 | 407 | -0.214 | <0.0001 |
|  | Role Function | 687 | -0.255 | <0.0001 | 277 | -0.320 | <0.0001 | 410 | -0.236 | <0.0001 |
|  | Emotional function | 687 | -0.218 | <0.0001 | 276 | -0.365 | <0.0001 | 411 | -0.133 | 0.007 |
|  | Cognitive function | 689 | -0.176 | <0.0001 | 277 | -0.350 | <0.0001 | 412 | -0.061 | 0.220 |
|  | Social Function | 674 | -0.177 | <0.0001 | 262 | -0.337 | <0.0001 | 412 | -0.086 | 0.082 |
| Hives, P† | Global QoL‡ | 686 | -0.108 | 0.005 | 275 | -0.177 | 0.003 | 411 | 0.028 | 0.574 |
|  | Physical Function | 681 | -0.080 | 0.037 | 274 | -0.231 | <0.0001 | 407 | 0.161 | 0.001 |
|  | Role Function | 686 | -0.137 | <0.0001 | 276 | -0.240 | <0.0001 | 410 | 0.008 | 0.871 |
|  | Emotional function | 686 | -0.108 | 0.005 | 275 | -0.212 | <0.0001 | 411 | 0.001 | 0.976 |
|  | Cognitive function | 688 | -0.167 | <0.0001 | 276 | -0.245 | <0.0001 | 412 | -0.071 | 0.151 |
|  | Social Function | 673 | -0.267 | <0.0001 | 261 | -0.302 | <0.0001 | 412 | -0.194 | <0.0001 |
| Hot flashes, F† | Global QoL‡ | 672 | -0.186 | <0.0001 | 272 | -0.160 | 0.008 | 400 | -0.203 | <0.0001 |
|  | Physical Function | 669 | -0.236 | <0.0001 | 271 | -0.242 | <0.0001 | 398 | -0.269 | <0.0001 |
|  | Role Function | 672 | -0.109 | 0.005 | 273 | -0.080 | 0.189 | 399 | -0.130 | 0.009 |
|  | Emotional function | 672 | -0.126 | 0.001 | 272 | -0.251 | <0.0001 | 400 | -0.061 | 0.225 |
|  | Cognitive function | 674 | -0.279 | <0.0001 | 273 | -0.300 | <0.0001 | 401 | -0.271 | <0.0001 |
|  | Social Function | 659 | -0.097 | 0.013 | 258 | -0.068 | 0.276 | 401 | -0.117 | 0.019 |
| Hot flashes, S† | Global QoL‡ | 679 | -0.191 | <0.0001 | 272 | -0.151 | 0.013 | 407 | -0.211 | <0.0001 |
|  | Physical Function | 675 | -0.230 | <0.0001 | 271 | -0.230 | <0.0001 | 404 | -0.255 | <0.0001 |
|  | Role Function | 679 | -0.111 | 0.004 | 273 | -0.075 | 0.219 | 406 | -0.130 | 0.009 |
|  | Emotional function | 679 | -0.116 | 0.003 | 272 | -0.248 | <0.0001 | 407 | -0.046 | 0.359 |
|  | Cognitive function | 681 | -0.265 | <0.0001 | 273 | -0.276 | <0.0001 | 408 | -0.262 | <0.0001 |
|  | Social Function | 666 | -0.106 | 0.006 | 258 | -0.071 | 0.258 | 408 | -0.125 | 0.011 |
| Increased sweating, F† | Global QoL‡ | 684 | -0.219 | <0.0001 | 273 | -0.184 | 0.002 | 411 | -0.263 | <0.0001 |
|  | Physical Function | 679 | -0.209 | <0.0001 | 272 | -0.257 | <0.0001 | 407 | -0.236 | <0.0001 |
|  | Role Function | 684 | -0.135 | <0.0001 | 274 | -0.130 | 0.031 | 410 | -0.155 | 0.002 |
|  | Emotional function | 684 | -0.158 | <0.0001 | 273 | -0.237 | <0.0001 | 411 | -0.120 | 0.015 |
|  | Cognitive function | 686 | -0.197 | <0.0001 | 274 | -0.252 | <0.0001 | 412 | -0.176 | <0.0001 |
|  | Social Function | 671 | -0.128 | 0.001 | 259 | -0.132 | 0.034 | 412 | -0.136 | 0.006 |
| Increased sweating, S† | Global QoL‡ | 685 | -0.208 | <0.0001 | 274 | -0.207 | 0.001 | 411 | -0.226 | <0.0001 |
|  | Physical Function | 680 | -0.207 | <0.0001 | 273 | -0.253 | <0.0001 | 407 | -0.232 | <0.0001 |
|  | Role Function | 685 | -0.141 | <0.0001 | 275 | -0.182 | 0.002 | 410 | -0.133 | 0.007 |
|  | Emotional function | 685 | -0.126 | 0.001 | 274 | -0.223 | <0.0001 | 411 | -0.074 | 0.135 |
|  | Cognitive function | 687 | -0.209 | <0.0001 | 275 | -0.224 | <0.0001 | 412 | -0.214 | <0.0001 |
|  | Social Function | 672 | -0.127 | 0.001 | 260 | -0.164 | 0.008 | 412 | -0.117 | 0.018 |
| Insomnia, S† | Global QoL‡ | 684 | -0.231 | <0.0001 | 273 | -0.079 | 0.192 | 411 | -0.301 | <0.0001 |
|  | Physical Function | 679 | -0.222 | <0.0001 | 272 | -0.210 | <0.0001 | 407 | -0.212 | <0.0001 |
|  | Role Function | 684 | -0.174 | <0.0001 | 274 | -0.055 | 0.364 | 410 | -0.241 | <0.0001 |
|  | Emotional function | 684 | -0.186 | <0.0001 | 273 | -0.141 | 0.019 | 411 | -0.206 | <0.0001 |
|  | Cognitive function | 686 | -0.236 | <0.0001 | 274 | -0.204 | 0.001 | 412 | -0.242 | <0.0001 |
|  | Social Function | 671 | -0.218 | <0.0001 | 259 | -0.146 | 0.019 | 412 | -0.251 | <0.0001 |
| Insomnia, I† | Global QoL‡ | 680 | -0.321 | <0.0001 | 269 | -0.227 | <0.0001 | 411 | -0.361 | <0.0001 |
|  | Physical Function | 675 | -0.291 | <0.0001 | 268 | -0.332 | <0.0001 | 407 | -0.255 | <0.0001 |
|  | Role Function | 680 | -0.210 | <0.0001 | 270 | -0.125 | 0.041 | 410 | -0.255 | <0.0001 |
|  | Emotional function | 680 | -0.272 | <0.0001 | 269 | -0.327 | <0.0001 | 411 | -0.235 | <0.0001 |
|  | Cognitive function | 682 | -0.307 | <0.0001 | 270 | -0.335 | <0.0001 | 412 | -0.281 | <0.0001 |
|  | Social Function | 667 | -0.250 | <0.0001 | 255 | -0.212 | 0.001 | 412 | -0.264 | <0.0001 |
| Itching, S† | Global QoL‡ | 685 | -0.179 | <0.0001 | 275 | -0.127 | 0.036 | 410 | -0.206 | <0.0001 |
|  | Physical Function | 681 | -0.190 | <0.0001 | 274 | -0.158 | 0.009 | 407 | -0.259 | <0.0001 |
|  | Role Function | 685 | -0.076 | 0.047 | 276 | 0.007 | 0.914 | 409 | -0.126 | 0.011 |
|  | Emotional function | 685 | -0.101 | 0.008 | 275 | -0.095 | 0.117 | 410 | -0.105 | 0.033 |
|  | Cognitive function | 687 | -0.321 | <0.0001 | 276 | -0.241 | <0.0001 | 411 | -0.377 | <0.0001 |
|  | Social Function | 672 | -0.163 | <0.0001 | 261 | -0.099 | 0.111 | 411 | -0.201 | <0.0001 |
| Joint pain, F† | Global QoL‡ | 686 | -0.186 | <0.0001 | 275 | -0.257 | <0.0001 | 411 | -0.091 | 0.066 |
|  | Physical Function | 681 | -0.265 | <0.0001 | 274 | -0.278 | <0.0001 | 407 | -0.189 | <0.0001 |
|  | Role Function | 686 | -0.135 | <0.0001 | 276 | -0.180 | 0.003 | 410 | -0.052 | 0.291 |
|  | Emotional function | 686 | -0.180 | <0.0001 | 275 | -0.207 | 0.001 | 411 | -0.144 | 0.003 |
|  | Cognitive function | 688 | -0.247 | <0.0001 | 276 | 0.042 | 0.492 | 412 | -0.385 | <0.0001 |
|  | Social Function | 673 | -0.222 | <0.0001 | 261 | -0.260 | <0.0001 | 412 | -0.175 | <0.0001 |
| Joint pain, S† | Global QoL‡ | 686 | -0.216 | <0.0001 | 275 | -0.282 | <0.0001 | 411 | -0.120 | 0.015 |
|  | Physical Function | 681 | -0.303 | <0.0001 | 274 | -0.271 | <0.0001 | 407 | -0.245 | <0.0001 |
|  | Role Function | 686 | -0.166 | <0.0001 | 276 | -0.233 | <0.0001 | 410 | -0.078 | 0.114 |
|  | Emotional function | 686 | -0.187 | <0.0001 | 275 | -0.234 | <0.0001 | 411 | -0.140 | 0.004 |
|  | Cognitive function | 688 | -0.259 | <0.0001 | 276 | 0.064 | 0.289 | 412 | -0.406 | <0.0001 |
|  | Social Function | 673 | -0.250 | <0.0001 | 261 | -0.307 | <0.0001 | 412 | -0.196 | <0.0001 |
| Joint pain, I† | Global QoL‡ | 686 | -0.250 | <0.0001 | 275 | -0.344 | <0.0001 | 411 | -0.135 | 0.006 |
|  | Physical Function | 681 | -0.363 | <0.0001 | 274 | -0.354 | <0.0001 | 407 | -0.265 | <0.0001 |
|  | Role Function | 686 | -0.201 | <0.0001 | 276 | -0.322 | <0.0001 | 410 | -0.093 | 0.061 |
|  | Emotional function | 686 | -0.182 | <0.0001 | 275 | -0.320 | <0.0001 | 411 | -0.106 | 0.032 |
|  | Cognitive function | 688 | -0.302 | <0.0001 | 276 | -0.025 | 0.677 | 412 | -0.407 | <0.0001 |
|  | Social Function | 673 | -0.263 | <0.0001 | 261 | -0.342 | <0.0001 | 412 | -0.197 | <0.0001 |
| Memory, S† | Global QoL‡ | 686 | -0.333 | <0.0001 | 275 | -0.452 | <0.0001 | 411 | -0.245 | <0.0001 |
|  | Physical Function | 681 | -0.440 | <0.0001 | 274 | -0.437 | <0.0001 | 407 | -0.444 | <0.0001 |
|  | Role Function | 686 | -0.324 | <0.0001 | 276 | -0.313 | <0.0001 | 410 | -0.324 | <0.0001 |
|  | Emotional function | 686 | -0.247 | <0.0001 | 275 | -0.490 | <0.0001 | 411 | -0.106 | 0.031 |
|  | Cognitive function | 688 | -0.784 | <0.0001 | 276 | -0.758 | <0.0001 | 412 | -0.799 | <0.0001 |
|  | Social Function | 673 | -0.345 | <0.0001 | 261 | -0.334 | <0.0001 | 412 | -0.349 | <0.0001 |
| Memory, I† | Global QoL‡ | 685 | -0.318 | <0.0001 | 275 | -0.443 | <0.0001 | 410 | -0.246 | <0.0001 |
|  | Physical Function | 679 | -0.351 | <0.0001 | 274 | -0.447 | <0.0001 | 405 | -0.328 | <0.0001 |
|  | Role Function | 684 | -0.223 | <0.0001 | 276 | -0.264 | <0.0001 | 408 | -0.210 | <0.0001 |
|  | Emotional function | 685 | -0.243 | <0.0001 | 275 | -0.474 | <0.0001 | 410 | -0.103 | 0.037 |
|  | Cognitive function | 686 | -0.641 | <0.0001 | 276 | -0.686 | <0.0001 | 410 | -0.616 | <0.0001 |
|  | Social Function | 671 | -0.258 | <0.0001 | 261 | -0.367 | <0.0001 | 410 | -0.206 | <0.0001 |
| Mouth/throat sores, S† | Global QoL‡ | 686 | -0.097 | 0.011 | 274 | -0.091 | 0.135 | 412 | -0.119 | 0.015 |
|  | Physical Function | 680 | -0.147 | <0.0001 | 273 | -0.030 | 0.622 | 407 | -0.295 | <0.0001 |
|  | Role Function | 685 | -0.116 | 0.002 | 275 | 0.068 | 0.262 | 410 | -0.255 | <0.0001 |
|  | Emotional function | 686 | -0.017 | 0.652 | 274 | -0.001 | 0.986 | 412 | -0.039 | 0.426 |
|  | Cognitive function | 687 | -0.173 | <0.0001 | 275 | 0.026 | 0.662 | 412 | -0.321 | <0.0001 |
|  | Social Function | 672 | -0.119 | 0.002 | 260 | 0.040 | 0.521 | 412 | -0.242 | <0.0001 |
| Mouth/throat sores, I† | Global QoL‡ | 687 | -0.116 | 0.002 | 273 | -0.097 | 0.109 | 414 | -0.135 | 0.006 |
|  | Physical Function | 682 | -0.170 | <0.0001 | 272 | -0.058 | 0.339 | 410 | -0.274 | <0.0001 |
|  | Role Function | 687 | -0.146 | <0.0001 | 274 | 0.005 | 0.935 | 413 | -0.237 | <0.0001 |
|  | Emotional function | 687 | -0.059 | 0.119 | 273 | -0.059 | 0.332 | 414 | -0.064 | 0.193 |
|  | Cognitive function | 689 | -0.176 | <0.0001 | 274 | -0.025 | 0.683 | 415 | -0.274 | <0.0001 |
|  | Social Function | 674 | -0.133 | 0.001 | 259 | 0.033 | 0.601 | 415 | -0.238 | <0.0001 |
| Muscle pain, F† | Global QoL‡ | 686 | -0.353 | <0.0001 | 275 | -0.313 | <0.0001 | 411 | -0.329 | <0.0001 |
|  | Physical Function | 681 | -0.414 | <0.0001 | 274 | -0.360 | <0.0001 | 407 | -0.388 | <0.0001 |
|  | Role Function | 686 | -0.275 | <0.0001 | 276 | -0.228 | <0.0001 | 410 | -0.257 | <0.0001 |
|  | Emotional function | 686 | -0.205 | <0.0001 | 275 | -0.323 | <0.0001 | 411 | -0.128 | 0.009 |
|  | Cognitive function | 688 | -0.396 | <0.0001 | 276 | -0.281 | <0.0001 | 412 | -0.448 | <0.0001 |
|  | Social Function | 673 | -0.294 | <0.0001 | 261 | -0.210 | 0.001 | 412 | -0.310 | <0.0001 |
| Muscle pain, S† | Global QoL‡ | 686 | -0.376 | <0.0001 | 275 | -0.352 | <0.0001 | 411 | -0.335 | <0.0001 |
|  | Physical Function | 681 | -0.437 | <0.0001 | 274 | -0.355 | <0.0001 | 407 | -0.409 | <0.0001 |
|  | Role Function | 686 | -0.297 | <0.0001 | 276 | -0.261 | <0.0001 | 410 | -0.271 | <0.0001 |
|  | Emotional function | 686 | -0.183 | <0.0001 | 275 | -0.297 | <0.0001 | 411 | -0.105 | 0.034 |
|  | Cognitive function | 688 | -0.379 | <0.0001 | 276 | -0.231 | <0.0001 | 412 | -0.438 | <0.0001 |
|  | Social Function | 673 | -0.309 | <0.0001 | 261 | -0.276 | <0.0001 | 412 | -0.292 | <0.0001 |
| Muscle pain, I† | Global QoL‡ | 685 | -0.365 | <0.0001 | 274 | -0.380 | <0.0001 | 411 | -0.303 | <0.0001 |
|  | Physical Function | 680 | -0.459 | <0.0001 | 273 | -0.419 | <0.0001 | 407 | -0.400 | <0.0001 |
|  | Role Function | 685 | -0.303 | <0.0001 | 275 | -0.267 | <0.0001 | 410 | -0.274 | <0.0001 |
|  | Emotional function | 685 | -0.145 | <0.0001 | 274 | -0.276 | <0.0001 | 411 | -0.069 | 0.161 |
|  | Cognitive function | 687 | -0.372 | <0.0001 | 275 | -0.268 | <0.0001 | 412 | -0.411 | <0.0001 |
|  | Social Function | 672 | -0.318 | <0.0001 | 260 | -0.355 | <0.0001 | 412 | -0.274 | <0.0001 |
| Nausea, F† | Global QoL‡ | 688 | -0.412 | <0.0001 | 275 | -0.545 | <0.0001 | 413 | -0.357 | <0.0001 |
|  | Physical Function | 683 | -0.229 | <0.0001 | 274 | -0.356 | <0.0001 | 409 | -0.213 | <0.0001 |
|  | Role Function | 688 | -0.309 | <0.0001 | 276 | -0.476 | <0.0001 | 412 | -0.241 | <0.0001 |
|  | Emotional function | 688 | -0.249 | <0.0001 | 275 | -0.296 | <0.0001 | 413 | -0.221 | <0.0001 |
|  | Cognitive function | 690 | -0.222 | <0.0001 | 276 | -0.236 | <0.0001 | 414 | -0.228 | <0.0001 |
|  | Social Function | 675 | -0.231 | <0.0001 | 261 | -0.398 | <0.0001 | 414 | -0.150 | 0.002 |
| Nausea, S† | Global QoL‡ | 689 | -0.408 | <0.0001 | 276 | -0.517 | <0.0001 | 413 | -0.364 | <0.0001 |
|  | Physical Function | 684 | -0.225 | <0.0001 | 275 | -0.319 | <0.0001 | 409 | -0.229 | <0.0001 |
|  | Role Function | 689 | -0.313 | <0.0001 | 277 | -0.444 | <0.0001 | 412 | -0.268 | <0.0001 |
|  | Emotional function | 689 | -0.224 | <0.0001 | 276 | -0.262 | <0.0001 | 413 | -0.202 | <0.0001 |
|  | Cognitive function | 691 | -0.223 | <0.0001 | 277 | -0.199 | 0.001 | 414 | -0.252 | <0.0001 |
|  | Social Function | 676 | -0.237 | <0.0001 | 262 | -0.370 | <0.0001 | 414 | -0.173 | <0.0001 |
| Numbness & tingling, S† | Global QoL‡ | 686 | 0.024 | 0.528 | 274 | 0.092 | 0.130 | 412 | 0.013 | 0.786 |
|  | Physical Function | 681 | -0.152 | <0.0001 | 273 | -0.054 | 0.370 | 408 | -0.193 | <0.0001 |
|  | Role Function | 686 | -0.076 | 0.047 | 275 | -0.003 | 0.958 | 411 | -0.108 | 0.028 |
|  | Emotional function | 686 | 0.106 | 0.006 | 274 | 0.098 | 0.105 | 412 | 0.109 | 0.026 |
|  | Cognitive function | 688 | -0.131 | 0.001 | 275 | -0.061 | 0.317 | 413 | -0.167 | 0.001 |
|  | Social Function | 673 | -0.025 | 0.514 | 260 | 0.041 | 0.510 | 413 | -0.049 | 0.318 |
| Numbness & tingling, I† | Global QoL‡ | 686 | -0.186 | <0.0001 | 274 | -0.155 | 0.010 | 412 | -0.147 | 0.003 |
|  | Physical Function | 681 | -0.286 | <0.0001 | 273 | -0.183 | 0.002 | 408 | -0.288 | <0.0001 |
|  | Role Function | 686 | -0.161 | <0.0001 | 275 | -0.007 | 0.909 | 411 | -0.195 | <0.0001 |
|  | Emotional function | 686 | -0.038 | 0.318 | 274 | -0.107 | 0.077 | 412 | 0.001 | 0.987 |
|  | Cognitive function | 688 | -0.283 | <0.0001 | 275 | -0.288 | <0.0001 | 413 | -0.269 | <0.0001 |
|  | Social Function | 673 | -0.153 | <0.0001 | 260 | -0.121 | 0.052 | 413 | -0.144 | 0.003 |
| Pain, F† | Global QoL‡ | 686 | -0.500 | <0.0001 | 275 | -0.281 | <0.0001 | 411 | -0.512 | <0.0001 |
|  | Physical Function | 681 | -0.425 | <0.0001 | 274 | -0.414 | <0.0001 | 407 | -0.305 | <0.0001 |
|  | Role Function | 686 | -0.434 | <0.0001 | 276 | -0.353 | <0.0001 | 410 | -0.393 | <0.0001 |
|  | Emotional function | 686 | -0.293 | <0.0001 | 275 | -0.246 | <0.0001 | 411 | -0.276 | <0.0001 |
|  | Cognitive function | 688 | -0.291 | <0.0001 | 276 | -0.041 | 0.493 | 412 | -0.390 | <0.0001 |
|  | Social Function | 673 | -0.406 | <0.0001 | 261 | -0.332 | <0.0001 | 412 | -0.371 | <0.0001 |
| Pain, S† | Global QoL‡ | 686 | -0.504 | <0.0001 | 275 | -0.336 | <0.0001 | 411 | -0.497 | <0.0001 |
|  | Physical Function | 681 | -0.428 | <0.0001 | 274 | -0.406 | <0.0001 | 407 | -0.320 | <0.0001 |
|  | Role Function | 686 | -0.419 | <0.0001 | 276 | -0.353 | <0.0001 | 410 | -0.382 | <0.0001 |
|  | Emotional function | 686 | -0.259 | <0.0001 | 275 | -0.269 | <0.0001 | 411 | -0.203 | <0.0001 |
|  | Cognitive function | 688 | -0.280 | <0.0001 | 276 | -0.049 | 0.414 | 412 | -0.367 | <0.0001 |
|  | Social Function | 673 | -0.399 | <0.0001 | 261 | -0.364 | <0.0001 | 412 | -0.349 | <0.0001 |
| Pain, I† | Global QoL‡ | 685 | -0.512 | <0.0001 | 275 | -0.496 | <0.0001 | 410 | -0.452 | <0.0001 |
|  | Physical Function | 680 | -0.462 | <0.0001 | 274 | -0.514 | <0.0001 | 406 | -0.322 | <0.0001 |
|  | Role Function | 685 | -0.441 | <0.0001 | 276 | -0.416 | <0.0001 | 409 | -0.397 | <0.0001 |
|  | Emotional function | 685 | -0.275 | <0.0001 | 275 | -0.444 | <0.0001 | 410 | -0.172 | <0.0001 |
|  | Cognitive function | 687 | -0.313 | <0.0001 | 276 | -0.269 | <0.0001 | 411 | -0.322 | <0.0001 |
|  | Social Function | 672 | -0.420 | <0.0001 | 261 | -0.473 | <0.0001 | 411 | -0.350 | <0.0001 |
| Pain and swelling at injection site, P† | Global QoL‡ | 682 | -0.004 | 0.911 | 274 | -0.081 | 0.181 | 408 | 0.148 | 0.003 |
|  | Physical Function | 677 | 0.002 | 0.958 | 273 | -0.096 | 0.114 | 404 | 0.214 | <0.0001 |
|  | Role Function | 682 | -0.023 | 0.547 | 275 | -0.111 | 0.066 | 407 | 0.133 | 0.007 |
|  | Emotional function | 682 | 0.041 | 0.284 | 274 | -0.118 | 0.052 | 408 | 0.191 | <0.0001 |
|  | Cognitive function | 684 | -0.074 | 0.053 | 275 | -0.204 | 0.001 | 409 | 0.068 | 0.169 |
|  | Social Function | 669 | -0.060 | 0.118 | 260 | -0.152 | 0.014 | 409 | 0.068 | 0.167 |
| Painful urination, S† | Global QoL‡ | 661 | 0.000 | 0.998 | 257 | -0.027 | 0.671 | 404 | -0.056 | 0.260 |
|  | Physical Function | 656 | 0.169 | <0.0001 | 256 | 0.106 | 0.089 | 400 | 0.108 | 0.032 |
|  | Role Function | 661 | 0.043 | 0.264 | 258 | 0.031 | 0.620 | 403 | -0.008 | 0.880 |
|  | Emotional function | 661 | 0.008 | 0.842 | 257 | -0.056 | 0.375 | 404 | 0.034 | 0.491 |
|  | Cognitive function | 663 | 0.057 | 0.140 | 258 | 0.063 | 0.311 | 405 | 0.029 | 0.555 |
|  | Social Function | 662 | 0.042 | 0.280 | 257 | 0.018 | 0.769 | 405 | 0.004 | 0.935 |
| Rash, P† | Global QoL‡ | 687 | -0.102 | 0.007 | 275 | -0.075 | 0.216 | 412 | -0.062 | 0.210 |
|  | Physical Function | 682 | -0.085 | 0.026 | 274 | -0.168 | 0.005 | 408 | 0.061 | 0.222 |
|  | Role Function | 687 | -0.118 | 0.002 | 276 | -0.155 | 0.010 | 411 | -0.037 | 0.458 |
|  | Emotional function | 687 | -0.118 | 0.002 | 275 | -0.100 | 0.097 | 412 | -0.122 | 0.013 |
|  | Cognitive function | 689 | -0.192 | <0.0001 | 276 | -0.167 | 0.005 | 413 | -0.191 | <0.0001 |
|  | Social Function | 674 | -0.283 | <0.0001 | 261 | -0.218 | <0.0001 | 413 | -0.292 | <0.0001 |
| Ringing in ears, S† | Global QoL‡ | 686 | -0.108 | 0.005 | 275 | -0.322 | <0.0001 | 411 | -0.057 | 0.251 |
|  | Physical Function | 681 | 0.052 | 0.173 | 274 | -0.151 | 0.012 | 407 | 0.043 | 0.386 |
|  | Role Function | 686 | -0.038 | 0.317 | 276 | -0.167 | 0.005 | 410 | -0.042 | 0.397 |
|  | Emotional function | 686 | -0.118 | 0.002 | 275 | -0.292 | <0.0001 | 411 | -0.052 | 0.289 |
|  | Cognitive function | 688 | -0.066 | 0.083 | 276 | -0.232 | <0.0001 | 412 | 0.001 | 0.977 |
|  | Social Function | 673 | -0.021 | 0.583 | 261 | -0.218 | <0.0001 | 412 | 0.033 | 0.507 |
| Sad, F† | Global QoL‡ | 683 | -0.577 | <0.0001 | 272 | -0.582 | <0.0001 | 411 | -0.552 | <0.0001 |
|  | Physical Function | 678 | -0.368 | <0.0001 | 271 | -0.411 | <0.0001 | 407 | -0.285 | <0.0001 |
|  | Role Function | 683 | -0.361 | <0.0001 | 273 | -0.318 | <0.0001 | 410 | -0.341 | <0.0001 |
|  | Emotional function | 683 | -0.711 | <0.0001 | 272 | -0.621 | <0.0001 | 411 | -0.751 | <0.0001 |
|  | Cognitive function | 685 | -0.315 | <0.0001 | 273 | -0.385 | <0.0001 | 412 | -0.259 | <0.0001 |
|  | Social Function | 670 | -0.480 | <0.0001 | 258 | -0.421 | <0.0001 | 412 | -0.488 | <0.0001 |
| Sad, S† | Global QoL‡ | 683 | -0.572 | <0.0001 | 272 | -0.546 | <0.0001 | 411 | -0.571 | <0.0001 |
|  | Physical Function | 678 | -0.369 | <0.0001 | 271 | -0.402 | <0.0001 | 407 | -0.301 | <0.0001 |
|  | Role Function | 683 | -0.378 | <0.0001 | 273 | -0.323 | <0.0001 | 410 | -0.372 | <0.0001 |
|  | Emotional function | 683 | -0.692 | <0.0001 | 272 | -0.601 | <0.0001 | 411 | -0.733 | <0.0001 |
|  | Cognitive function | 685 | -0.307 | <0.0001 | 273 | -0.357 | <0.0001 | 412 | -0.259 | <0.0001 |
|  | Social Function | 670 | -0.473 | <0.0001 | 258 | -0.429 | <0.0001 | 412 | -0.478 | <0.0001 |
| Sad, I† | Global QoL‡ | 683 | -0.520 | <0.0001 | 272 | -0.511 | <0.0001 | 411 | -0.500 | <0.0001 |
|  | Physical Function | 678 | -0.393 | <0.0001 | 271 | -0.512 | <0.0001 | 407 | -0.267 | <0.0001 |
|  | Role Function | 683 | -0.358 | <0.0001 | 273 | -0.358 | <0.0001 | 410 | -0.322 | <0.0001 |
|  | Emotional function | 683 | -0.573 | <0.0001 | 272 | -0.574 | <0.0001 | 411 | -0.558 | <0.0001 |
|  | Cognitive function | 685 | -0.279 | <0.0001 | 273 | -0.401 | <0.0001 | 412 | -0.193 | <0.0001 |
|  | Social Function | 670 | -0.419 | <0.0001 | 258 | -0.496 | <0.0001 | 412 | -0.361 | <0.0001 |
| Shortness of breath, S† | Global QoL‡ | 688 | -0.492 | <0.0001 | 276 | -0.497 | <0.0001 | 412 | -0.448 | <0.0001 |
|  | Physical Function | 683 | -0.543 | <0.0001 | 275 | -0.461 | <0.0001 | 408 | -0.562 | <0.0001 |
|  | Role Function | 688 | -0.504 | <0.0001 | 277 | -0.394 | <0.0001 | 411 | -0.548 | <0.0001 |
|  | Emotional function | 688 | -0.331 | <0.0001 | 276 | -0.478 | <0.0001 | 412 | -0.254 | <0.0001 |
|  | Cognitive function | 690 | -0.429 | <0.0001 | 277 | -0.419 | <0.0001 | 413 | -0.402 | <0.0001 |
|  | Social Function | 675 | -0.473 | <0.0001 | 262 | -0.453 | <0.0001 | 413 | -0.459 | <0.0001 |
| Shortness of breath, I† | Global QoL‡ | 686 | -0.519 | <0.0001 | 275 | -0.528 | <0.0001 | 411 | -0.475 | <0.0001 |
|  | Physical Function | 681 | -0.572 | <0.0001 | 274 | -0.521 | <0.0001 | 407 | -0.575 | <0.0001 |
|  | Role Function | 686 | -0.514 | <0.0001 | 276 | -0.368 | <0.0001 | 410 | -0.572 | <0.0001 |
|  | Emotional function | 686 | -0.336 | <0.0001 | 275 | -0.485 | <0.0001 | 411 | -0.268 | <0.0001 |
|  | Cognitive function | 688 | -0.443 | <0.0001 | 276 | -0.486 | <0.0001 | 412 | -0.400 | <0.0001 |
|  | Social Function | 673 | -0.489 | <0.0001 | 261 | -0.501 | <0.0001 | 412 | -0.463 | <0.0001 |
| Swelling of arms or legs, F† | Global QoL‡ | 687 | -0.143 | <0.0001 | 276 | 0.026 | 0.664 | 411 | -0.233 | <0.0001 |
|  | Physical Function | 683 | -0.241 | <0.0001 | 275 | -0.178 | 0.003 | 408 | -0.318 | <0.0001 |
|  | Role Function | 687 | -0.254 | <0.0001 | 277 | -0.175 | 0.003 | 410 | -0.302 | <0.0001 |
|  | Emotional function | 687 | -0.095 | 0.013 | 276 | -0.063 | 0.301 | 411 | -0.110 | 0.025 |
|  | Cognitive function | 689 | -0.181 | <0.0001 | 277 | -0.171 | 0.004 | 412 | -0.177 | <0.0001 |
|  | Social Function | 674 | -0.177 | <0.0001 | 262 | 0.016 | 0.795 | 412 | -0.262 | <0.0001 |
| Swelling of arms or legs, S† | Global QoL‡ | 687 | -0.159 | <0.0001 | 276 | -0.026 | 0.664 | 411 | -0.230 | <0.0001 |
|  | Physical Function | 683 | -0.245 | <0.0001 | 275 | -0.191 | 0.001 | 408 | -0.316 | <0.0001 |
|  | Role Function | 687 | -0.259 | <0.0001 | 277 | -0.206 | 0.001 | 410 | -0.293 | <0.0001 |
|  | Emotional function | 687 | -0.111 | 0.003 | 276 | -0.078 | 0.119 | 411 | -0.131 | 0.008 |
|  | Cognitive function | 689 | -0.172 | <0.0001 | 277 | -0.158 | 0.009 | 412 | -0.170 | 0.001 |
|  | Social Function | 674 | -0.173 | <0.0001 | 262 | -0.002 | 0.975 | 412 | -0.251 | <0.0001 |
| Swelling of arms or legs, I† | Global QoL‡ | 685 | -0.199 | <0.0001 | 276 | -0.184 | 0.002 | 409 | -0.177 | <0.0001 |
|  | Physical Function | 681 | -0.274 | <0.0001 | 275 | -0.196 | 0.001 | 406 | -0.303 | <0.0001 |
|  | Role Function | 685 | -0.233 | <0.0001 | 277 | -0.105 | 0.082 | 408 | -0.278 | <0.0001 |
|  | Emotional function | 685 | -0.071 | 0.064 | 276 | -0.170 | 0.005 | 409 | -0.018 | 0.717 |
|  | Cognitive function | 687 | -0.233 | <0.0001 | 277 | -0.295 | <0.0001 | 410 | -0.189 | <0.0001 |
|  | Social Function | 672 | -0.219 | <0.0001 | 262 | -0.173 | 0.005 | 410 | -0.224 | <0.0001 |
| Taste changes, S† | Global QoL‡ | 690 | -0.273 | <0.0001 | 275 | -0.410 | <0.0001 | 415 | -0.190 | <0.0001 |
|  | Physical Function | 685 | -0.275 | <0.0001 | 274 | -0.487 | <0.0001 | 411 | -0.210 | <0.0001 |
|  | Role Function | 690 | -0.312 | <0.0001 | 276 | -0.436 | <0.0001 | 414 | -0.253 | <0.0001 |
|  | Emotional function | 690 | -0.271 | <0.0001 | 275 | -0.544 | <0.0001 | 415 | -0.102 | 0.039 |
|  | Cognitive function | 692 | -0.293 | <0.0001 | 276 | -0.389 | <0.0001 | 416 | -0.237 | <0.0001 |
|  | Social Function | 677 | -0.263 | <0.0001 | 261 | -0.317 | <0.0001 | 416 | -0.248 | <0.0001 |
| Urinary frequency, F† | Global QoL‡ | 655 | -0.050 | 0.204 | 256 | -0.035 | 0.580 | 399 | -0.133 | 0.008 |
|  | Physical Function | 650 | 0.047 | 0.231 | 255 | -0.007 | 0.913 | 395 | -0.026 | 0.607 |
|  | Role Function | 655 | 0.001 | 0.972 | 257 | -0.058 | 0.352 | 398 | -0.018 | 0.728 |
|  | Emotional function | 655 | -0.171 | <0.0001 | 256 | -0.166 | 0.008 | 399 | -0.189 | <0.0001 |
|  | Cognitive function | 657 | 0.007 | 0.852 | 257 | -0.053 | 0.397 | 400 | 0.027 | 0.585 |
|  | Social Function | 656 | -0.017 | 0.668 | 256 | -0.001 | 0.986 | 400 | -0.076 | 0.129 |
| Urinary frequency, I† | Global QoL‡ | 655 | -0.119 | 0.002 | 256 | -0.092 | 0.142 | 399 | -0.178 | <0.0001 |
|  | Physical Function | 650 | -0.075 | 0.055 | 255 | -0.174 | 0.005 | 395 | -0.063 | 0.212 |
|  | Role Function | 655 | -0.079 | 0.043 | 257 | -0.081 | 0.196 | 398 | -0.100 | 0.047 |
|  | Emotional function | 655 | -0.170 | <0.0001 | 256 | -0.154 | 0.013 | 399 | -0.195 | <0.0001 |
|  | Cognitive function | 657 | -0.030 | 0.442 | 257 | -0.143 | 0.022 | 400 | 0.041 | 0.419 |
|  | Social Function | 656 | -0.131 | 0.001 | 256 | -0.178 | 0.004 | 400 | -0.122 | 0.014 |
| Urinary incontinence, F† | Global QoL‡ | 654 | -0.053 | 0.175 | 257 | 0.038 | 0.546 | 397 | -0.122 | 0.015 |
|  | Physical Function | 648 | -0.066 | 0.091 | 256 | -0.018 | 0.768 | 392 | -0.094 | 0.062 |
|  | Role Function | 653 | 0.005 | 0.908 | 258 | 0.088 | 0.156 | 395 | -0.040 | 0.433 |
|  | Emotional function | 654 | -0.072 | 0.064 | 257 | 0.053 | 0.401 | 397 | -0.150 | 0.003 |
|  | Cognitive function | 655 | 0.020 | 0.608 | 258 | -0.010 | 0.869 | 397 | 0.042 | 0.408 |
|  | Social Function | 655 | -0.047 | 0.233 | 258 | 0.000 | 0.994 | 397 | -0.075 | 0.136 |
| Urinary incontinence, I† | Global QoL‡ | 652 | -0.050 | 0.205 | 256 | 0.043 | 0.495 | 396 | -0.128 | 0.011 |
|  | Physical Function | 646 | -0.036 | 0.360 | 255 | -0.013 | 0.834 | 391 | -0.064 | 0.203 |
|  | Role Function | 651 | 0.009 | 0.815 | 257 | 0.073 | 0.245 | 394 | -0.036 | 0.480 |
|  | Emotional function | 652 | -0.092 | 0.018 | 256 | 0.046 | 0.467 | 396 | -0.183 | <0.0001 |
|  | Cognitive function | 653 | -0.003 | 0.944 | 257 | -0.014 | 0.817 | 396 | 0.005 | 0.924 |
|  | Social Function | 653 | -0.046 | 0.236 | 257 | 0.001 | 0.982 | 396 | -0.082 | 0.101 |
| Urinary urgency, F† | Global QoL‡ | 655 | -0.052 | 0.182 | 255 | -0.054 | 0.391 | 400 | -0.089 | 0.074 |
|  | Physical Function | 650 | -0.002 | 0.966 | 254 | -0.041 | 0.514 | 396 | -0.043 | 0.398 |
|  | Role Function | 655 | -0.025 | 0.527 | 256 | -0.057 | 0.365 | 399 | -0.032 | 0.521 |
|  | Emotional function | 655 | -0.114 | 0.003 | 255 | -0.138 | 0.027 | 400 | -0.112 | 0.025 |
|  | Cognitive function | 657 | 0.029 | 0.454 | 256 | -0.028 | 0.655 | 401 | 0.049 | 0.332 |
|  | Social Function | 657 | -0.044 | 0.259 | 256 | -0.064 | 0.310 | 401 | -0.059 | 0.238 |
| Urinary urgency, I† | Global QoL‡ | 653 | -0.091 | 0.021 | 253 | -0.105 | 0.095 | 400 | -0.113 | 0.024 |
|  | Physical Function | 648 | -0.037 | 0.349 | 252 | -0.169 | 0.007 | 396 | 0.007 | 0.896 |
|  | Role Function | 653 | -0.028 | 0.477 | 254 | -0.083 | 0.189 | 399 | -0.006 | 0.907 |
|  | Emotional function | 653 | -0.153 | <0.0001 | 253 | -0.206 | 0.001 | 400 | -0.134 | 0.007 |
|  | Cognitive function | 655 | 0.013 | 0.747 | 254 | -0.133 | 0.035 | 401 | 0.100 | 0.046 |
|  | Social Function | 655 | -0.089 | 0.023 | 254 | -0.177 | 0.005 | 401 | -0.052 | 0.298 |
| Vomiting, F† | Global QoL‡ | 687 | -0.234 | <0.0001 | 276 | -0.159 | 0.008 | 411 | -0.276 | <0.0001 |
|  | Physical Function | 682 | -0.198 | <0.0001 | 275 | -0.088 | 0.145 | 407 | -0.274 | <0.0001 |
|  | Role Function | 687 | -0.183 | <0.0001 | 277 | -0.005 | 0.931 | 410 | -0.271 | <0.0001 |
|  | Emotional function | 687 | -0.042 | 0.268 | 276 | 0.022 | 0.713 | 411 | -0.069 | 0.162 |
|  | Cognitive function | 689 | -0.200 | <0.0001 | 277 | -0.078 | 0.195 | 412 | -0.258 | <0.0001 |
|  | Social Function | 674 | -0.227 | <0.0001 | 262 | -0.180 | 0.003 | 412 | -0.253 | <0.0001 |
| Vomiting, S† | Global QoL‡ | 688 | -0.222 | <0.0001 | 276 | -0.143 | 0.017 | 412 | -0.264 | <0.0001 |
|  | Physical Function | 683 | -0.193 | <0.0001 | 275 | -0.057 | 0.343 | 408 | -0.276 | <0.0001 |
|  | Role Function | 688 | -0.179 | <0.0001 | 277 | 0.028 | 0.640 | 411 | -0.277 | <0.0001 |
|  | Emotional function | 688 | -0.041 | 0.288 | 276 | 0.041 | 0.499 | 412 | -0.074 | 0.136 |
|  | Cognitive function | 690 | -0.198 | <0.0001 | 277 | -0.055 | 0.366 | 413 | -0.266 | <0.0001 |
|  | Social Function | 675 | -0.220 | <0.0001 | 262 | -0.167 | 0.007 | 413 | -0.248 | <0.0001 |

†F: Frequency item, S: Severity item, I: Interference item, A: Amount, P: Presence/absence. ‡QoL: Quality of life.
